# Discovery of V-0219: A Small-Molecule Positive Allosteric Modulator of the Glucagon-Like Peptide-1 Receptor toward Oral Treatment for “Diabesity”

**DOI:** 10.1021/acs.jmedchem.1c01842

**Published:** 2022-03-29

**Authors:** Juan M. Decara, Henar Vázquez-Villa, José Brea, Mónica Alonso, Raj Kamal Srivastava, Laura Orio, Francisco Alén, Juan Suárez, Elena Baixeras, Javier García-Cárceles, Andrea Escobar-Peña, Beat Lutz, Ramón Rodríguez, Eva Codesido, F. Javier Garcia-Ladona, Teresa A. Bennett, Juan A. Ballesteros, Jacobo Cruces, María I. Loza, Bellinda Benhamú, Fernando Rodríguez de Fonseca, María L. López-Rodríguez

**Affiliations:** † Unidad de Gestión Clínica de Salud Mental, Instituto IBIMA, Hospital Regional Universitario, Málaga E-29010, Spain; ‡ Departamento de Química Orgánica, Universidad Complutense de Madrid, Madrid E-28040, Spain; ∥ Biofarma Research group, USEF Screening Platform, CIMUS, USC, Santiago de Compostela E-15782, Spain; ⊥ Institute of Physiological Chemistry, University Medical Center of the Johannes Gutenberg, University of Mainz, Mainz 55128, Germany; ∇ Departamento de Psicobiología, Facultad de Psicología, Universidad Complutense de Madrid, Madrid E-28040, Spain; ○ Galchimia, O Pino, A Coruña E-15823, Spain; ◆ ABAXYS Therapeutics, Rue du Berceau, 91, Villers-la-Ville 1495, Belgium; ¶ ViviaBiotech S.L., Parque Científico de Madrid, Madrid E-28760, Spain

## Abstract

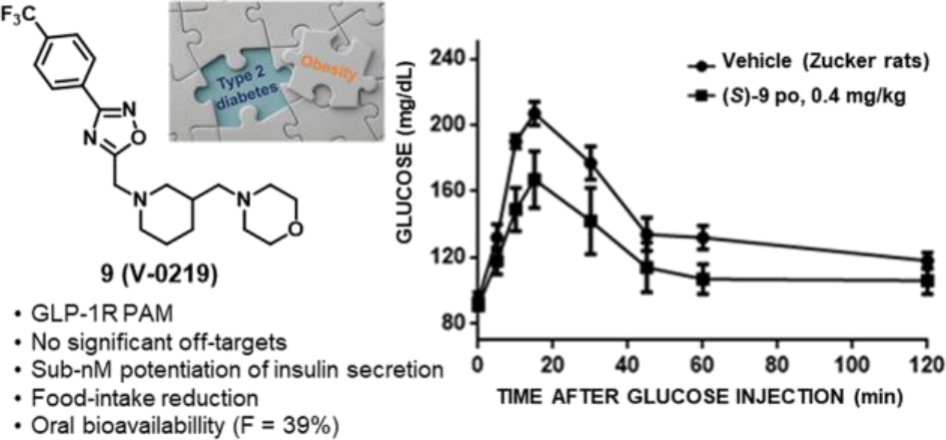

Peptidic agonists of the glucagon-like peptide-1 receptor (GLP-1R) have gained a prominent role in the therapy of type-2 diabetes and are being considered for reducing food intake in obesity. Potential advantages of small molecules acting as positive allosteric modulators (PAMs) of GLP-1R, including oral administration and reduced unwanted effects, could improve the utility of this class of drugs. Here, we describe the discovery of compound **9** (4-{[1-({3-[4-(trifluoromethyl)phenyl]-1,2,4-oxadiazol-5-yl}methyl)piperidin-3-yl]methyl}morpholine, V-0219) that exhibits enhanced efficacy of GLP-1R stimulation, subnanomolar potency in the potentiation of insulin secretion, and no significant off-target activities. The identified GLP-1R PAM shows a remarkable in vivo activity, reducing food intake and improving glucose handling in normal and diabetic rodents. Enantioselective synthesis revealed oral efficacy for (*S*)-**9** in animal models. Compound **9** behavior bolsters the interest of a small-molecule PAM of GLP-1R as a promising therapeutic approach for the increasingly prevalent obesity-associated diabetes.

## Introduction

Diabetes and obesity are serious public health concerns worldwide with increasing prevalence. “Diabesity”, the term for diabetes occurring in the context of obesity, is a newly evolving and fast growing epidemic of health impact worldwide.^1,2^ The lack of innovative therapies capable of preventing or effectively treating this syndrome is producing a severe impairment on the quality of human life and a substantial economic burden to the health care systems of the developed and developing countries. Diabetes is the resulting metabolic disorder of diminished production, bioavailability, and sensitivity of insulin against pathologically increased plasma glucose level. Current therapies for most common type 2 diabetes aim at normalizing glycemia and reducing the glycosylated hemoglobin HbA1c to 6%, with the long-term goal of preventing fatal consequences derived from the associated vascular damage. This is managed by means of multiple drugs aiming at reducing blood glucose or at increasing insulin secretion by the endocrine pancreas. However, most of the antidiabetic agents that are available in the market—biguanides (e.g., metformin), thiazolidinediones (e.g., pioglitazone), sulphonylureas (e.g., tolbutamide), meglitinides (e.g., repaglinide), and α-glucosidase inhibitors (e.g., acarbose)—lose efficacy over time, so combined treatments with more than one drug from the previous groups are used in the following phase. Despite all this, the treatment algorithms become complicated since diabetes is a disease that progresses, and a loss of pancreatic β-cell mass occurs, which leads to the need to treat with insulin. Therefore, the satisfactory management of glycemic control has remained a major clinical challenge in diabetic patients, and the alarmingly increasing incidence of type 2 diabetes worldwide has promoted the urgent need for novel therapeutic approaches. The discovery of glucagon-like peptide (GLP) and the development of therapeutic strategies aiming at favoring its physiological action have offered a possibility of addressing that unmet clinical need.^3^

GLP type 1 (GLP-1(7-36)-NH_2_, GLP-1) is a gastrointestinal peptide that is released by the cells of intestine and the α-cells of the endocrine pancreas in response to the elevation of certain nutrients in the plasma (for example, glucose). This peptide acts both in the free terminals of the peripheral sensory system innervating the intestine and in the insulin-secreting cells of the pancreatic islets of Langerhans. The actions of endogenous GLP-1 are threefold; on the one hand, it inhibits intestinal motility reducing the absorption of glucose and reduces intake by means of a net appetite decrease, and on the other hand, GLP-1 enhances glucose-dependent insulin release.^4^ This selectivity of action defines a special subtype of physiological signals that are generically referred to as incretins^5^ and are defined as those signals capable of increasing insulin release only in hyperglycemic conditions.^3^ In other words, endogenous GLP-1 acts only at high circulating glucose levels. However, the half-life time of this native peptide in blood is very low (1–2 min) as it is rapidly degraded mainly by the circulating dipeptidyl peptidase (DPP-4). Based on this principle, two alternative classes of antidiabetic drugs have been recently approved: DPP-4 inhibitors, which avoid the degradation of endogenous GLP-1,^3,6^ for example, vildagliptin (Galvus, Novartis),^7^ sitagliptin (Januvia, Merck),^8^ saxagliptin (Onglyza, Bristol-Myers-Squibb, and AstraZeneca),^9^ and linagliptin (Tradjenta, Boehringer Ingelheim),^10^ and synthetic GLP-1 peptide analogues^11,12^ with improved stability and duration of action that are agonists of the GLP-1R, a class B G-protein coupled receptor (GPCR) responsible for GLP-1-derived actions—e.g., exenatide (Byetta, AstraZeneca),^13^ liraglutide (Victoza, Novo Nordisk),^14^ albiglutide (Eperzan in Europe and Tanzeum in the US, GlaxoSmithKline),^15^ dulaglutide (Trulicity, Eli Lilly),^16^ lixisenatide (Lyxumia, Sanofi Aventis),^17^ and semaglutide (Ozempic, Novo Nordisk).^18^ These drugs capable of either increasing endogenous GLP-1 levels by preventing its degradation (DPP-4 inhibitors) or improving internal GLP-1 signaling (GLP-1R agonists) cover the need to treat those diabetic patients where the drugs used in the traditional algorithms fail. Overall, these drugs are insulin-releasing agents that do not produce unadvertised hypoglycemia episodes. Moreover, GLP-1R agonists have the capacity to reduce body weight and prevent the obesity-associated diabetes type 2, as demonstrated with the clinical use of liraglutide as an adjunct to a reduced-calorie diet and increased physical activity for the long-term management of body weight in adult overweight patients.

**Figure 1:**
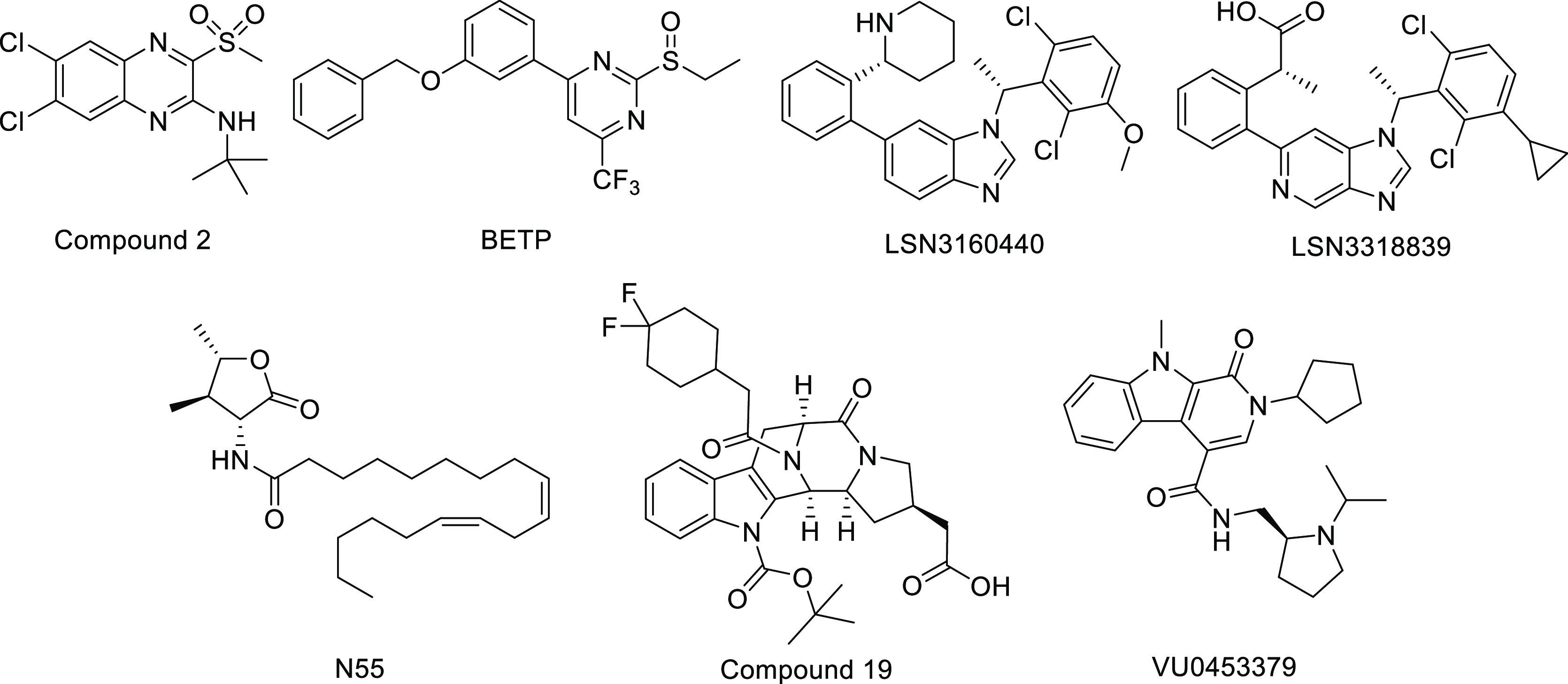
Representative small molecules reported as allosteric modulators of the GLP-1R.

The highly potent medicinal performance of peptide-based GLP-1 therapy in the treatment of type 2 diabetes, currently administered by injection except for oral semaglutide, has stimulated the search for more convenient, orally administered drugs. Hence, there is significant interest in the identification and development of nonpeptide agonists and PAMs of the GLP-1R^19^ with enhanced bioavailability, particularly oral absorption. In the present study, we have addressed GLP-1R activation via allosteric modulation, which is now a well-established paradigm for GPCR targets that provides both challenges and advantages for drug discovery over classic orthosteric ligands. Drugs that target a GLP-1R allosteric site may improve receptor specificity, thereby reducing side effects. Most of the allosteric modulators of the GLP-1R that have been described (Figure 1) display suboptimal potency and/or pharmacokinetics.^20−25^ Recently, a program toward optimization of LSN3160440 has succeeded in the discovery of structurally related LSN3318839 as an orally efficacious GLP-1 modulator.^26^ Herein, we report the discovery of the small molecule V-0219 (compound **9**), a very potent PAM of the GLP-1R that doubles insulin secretion at nanomolar concentrations and shows oral activity in rodent models of food intake and glucose handling.

## Results and Discussion

### Identification of Compound **9** as a PAM of the GLP-1R

**Figure 2:**
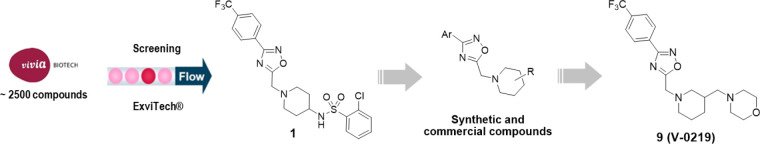
High-throughput screening of Vivia Biotech chemical library led to the identification of hit compound **1**. Subsequent functional evaluation of synthetic and commercial libraries of related oxadiazole derivatives allowed to identify compound **9** as a PAM of the GLP-1R.

**Figure 3:**
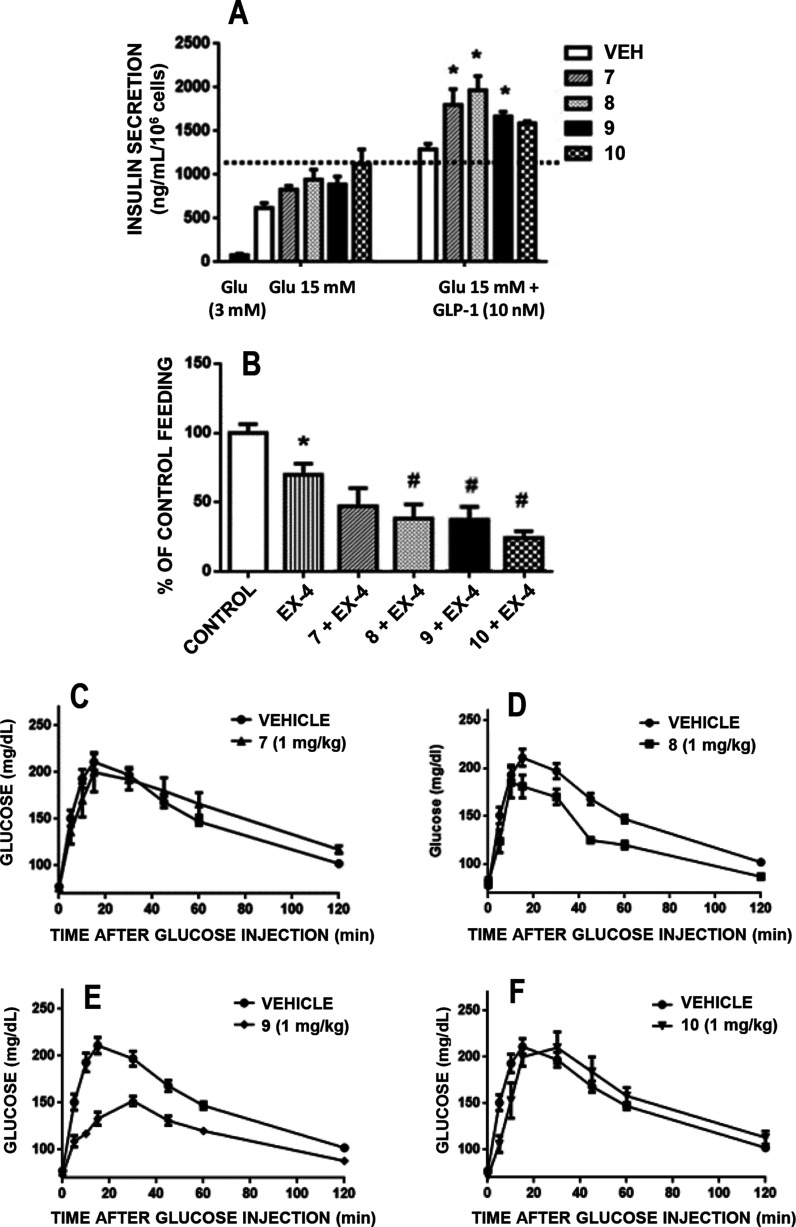
Functional screening of compounds **7–10** led to the selection of **9** for validation as PAM of the GLP-1R. (A) In vitro effects of compounds at a concentration of 1 μM on GLP-1 (10 nM) potentiation of high glucose (15 mM)-dependent insulin release by rat INS-1 β-cells (data are means of 4–6 replicate measures). * *P* < 0.05 versus same treatment without GLP-1. (B) Effects of compounds (1 μg/kg injected in 5 μL, icv) on the inhibition of feeding behavior induced by the GLP-1R agonist exendin-4 (100 ng per injection in 5 μL icv) in 12-h fasted male Wistar rats (*N* = 8 animals per group). * *P* < 0.05 versus vehicle. # *P* < 0.05 versus exendin-4 group. (C–F) Effect of the tested compound (1 mg/kg, ip) on plasma glucose levels after an ip 2 g/kg glucose load in 12-h fasted Wistar rats (*N* = 8 animals per group).

The proprietary ExviTech platform developed by Vivia Biotech is a highly sensitive method based on flow cytometry to assess activation of GPCRs, as described in previous reports.^27^ A functional whole cell assay is used to assess activity by measuring calcium mobilization in response to receptor activation. For screening, the tested compounds were added to HEK-293 cells stably expressing human GLP-1R, and after 10 min, 25% of the maximal effective concentration (EC_25_) of GLP-1 was added. The resulting percentage of cells that responded was then determined. The response for each well (i.e., compound) was compared to the control receiving only an EC_25_ concentration of GLP-1; any response above this level indicated a potential modulator. A Vivia Biotech chemical library of approximately 2500 compounds was screened on the ExviTech platform at a concentration of 10 μM. Oxadiazole derivative **1**, identified as a putative modulator of the GLP-1R, was considered a starting hit compound (Figure 2). A series of compounds related to **1** that contain different sulfonamide groups as the R substituent in the piperidine ring were synthesized (Figure 2, Table S1). Further structural diversity of the piperidine substituent was also explored, and analogues related to **1** were acquired from commercial libraries (Figure 2, Table S2). The generated compounds were evaluated at 10 μM for their ability to stimulate the GLP-1R in a cAMP assay in the presence of GLP-1, and the data for compounds **2–10** exhibiting a remarkable potentiation of the effect of GLP-1 (>30%) are displayed in Table 1. Among them, the most active analogues **7–10** were further assayed in vitro for their ability to potentiate GLP-1 (10 nM)-induced insulin secretion in INS-1 β-cells. INS-1 insulinoma cell line is a rat β-cell line that retains the ability of secreting insulin in a glucose-dependent manner. At 3 mM glucose, insulin secretion is low, and at 15 mM glucose, insulin secretion is boosted. Using this property and according to the data shown in Figure 3A, **7–10** were considered allosteric potentiators of the GLP-1R, being capable of enhancing GLP-1-induced secretion at 1 μM concentration, and selected for in vivo studies. The compounds were assessed for their ability to potentiate the feeding suppression induced by the GLP-1R agonist exendin-4 (Figure 3B) and their capacity to improve glucose handling in a glucose load test (Figure 3C–F). The selected compounds were found to display in vitro and in vivo activity as PAMs at GLP-1R. Altogether, **9** was found to be the most efficient one, being capable of (i) boosting glucose-dependent GLP-1-induced secretion of insulin from β-cells (Figure 3A); (ii) enhancing satiety induced by the GLP-1R agonist exendin-4 (Figure 3B); and (iii) inducing the most optimal response to glucose load, reducing the time spent in hyperglycemia in Wistar rats (Figure 3E).

**Table 1:**
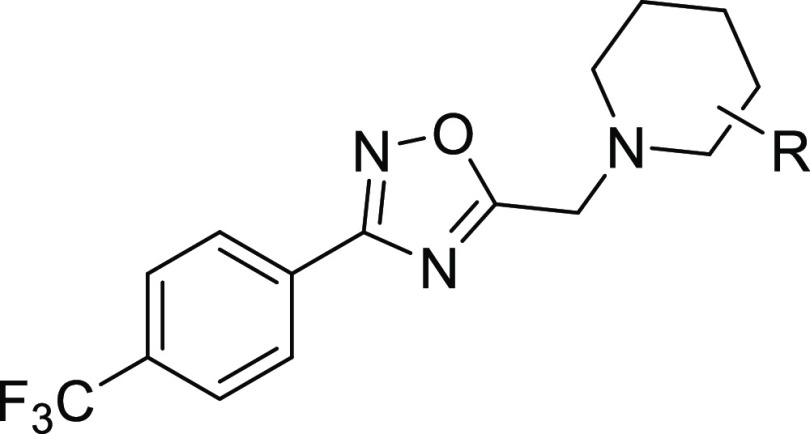

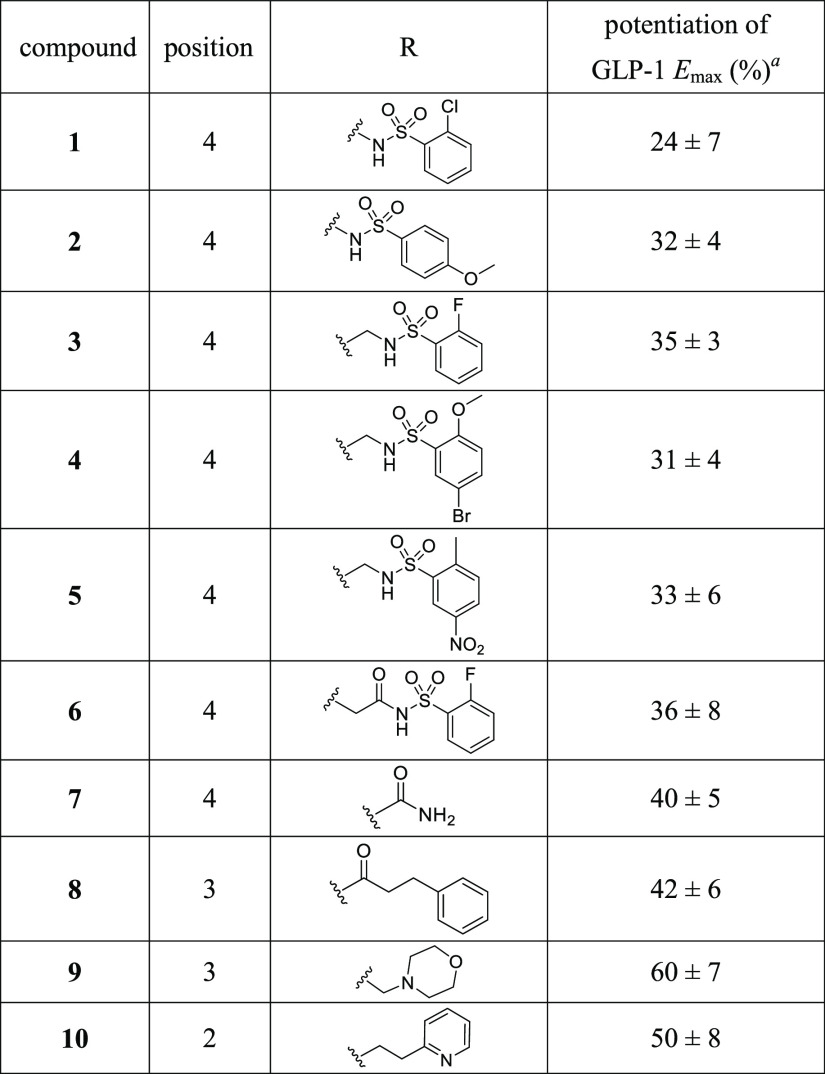
Identified PAMs of the GLP-1R^a^

a Potentiation of GLP-1 *E*_max_ at a fixed concentration of compound = 10 μM; values are the mean ± SEM of two experiments.

Accordingly, new analogues were synthesized maintaining the morpholin-4-ylmethyl fragment in the piperidine ring present in compound **9**, while the aryl moiety linked to the oxadiazole ring was modified (Figure 2, Table S3). None of the different substituents—i.e., methyl, methoxy, halogen, amino, methoxycarbonyl, carbamoyl—introduced at different positions in the phenyl ring afforded a potentiation of the GLP-1 effect higher than **9** in the cAMP assay.

### Chemistry

**Scheme 1:**
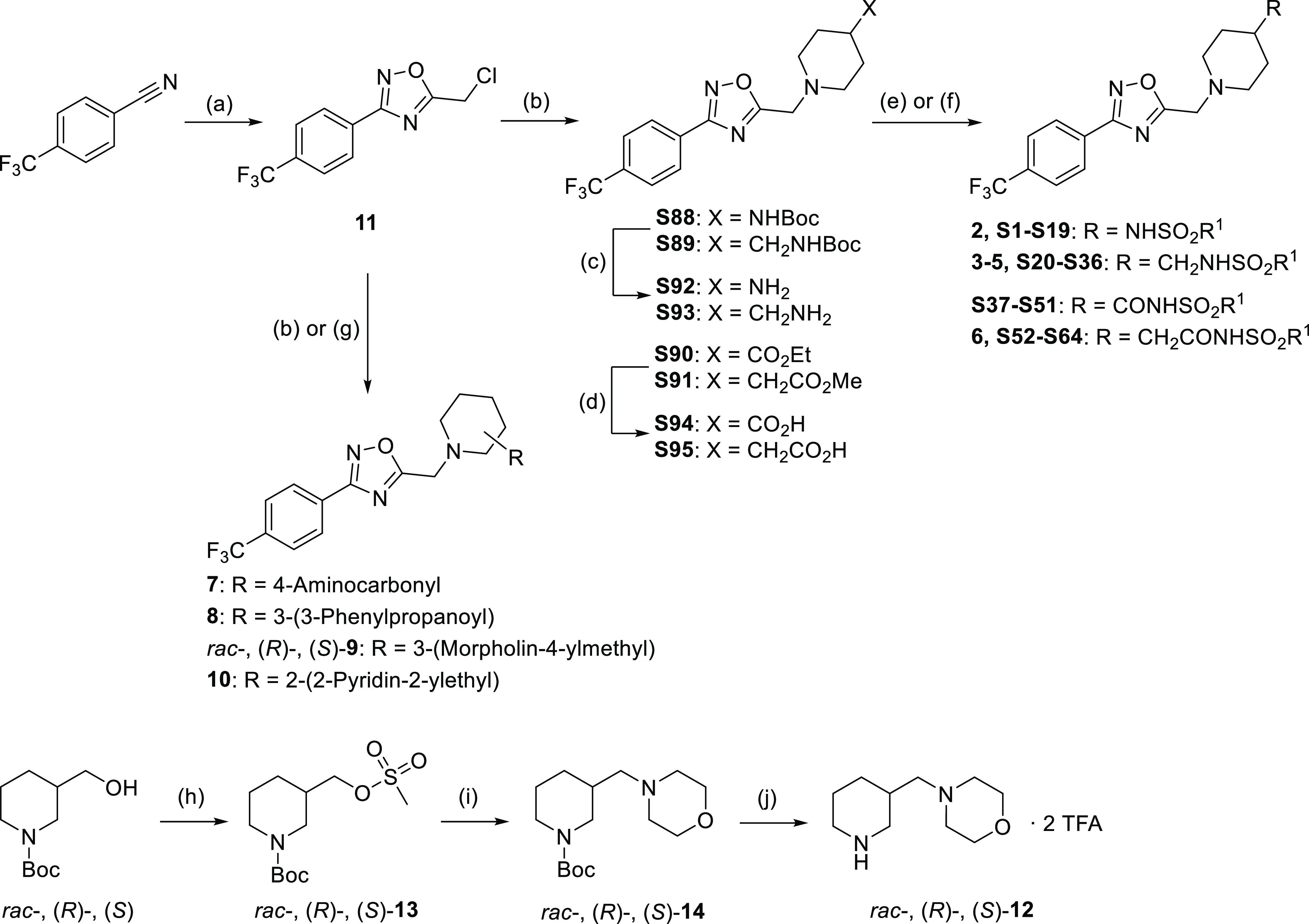
Synthesis of Compounds **2–10** and **S1–S64** Reagents and conditions: (a) (i) hydroxylamine hydrochloride, NaHCO_3_, ethanol, reflux, 16 h, 99%, (ii) chloroacetyl chloride, pyridine, 1,2-dichloroethane, reflux, 4 h, 48%; (b) *tert*-butyl piperidin-4-ylcarbamate, *tert*-butyl (piperidin-4-ylmethyl)carbamate, ethyl piperidine-4-carboxylate, methyl piperidin-4-ylacetate, piperidine-4-carboxamide, 3-phenyl-1-piperidin-3-ylpropan-1-one, or 2-(2-piperidin-2-ylethyl)pyridine, Cs_2_CO_3_, cat. NaI, ACN, reflux, 4–12 h, 57–96%; (c) 4 M HCl in dioxane, 50 °C, 12 h, 71–86%; (d) LiOH·H_2_O, THF/EtOH/H_2_O, rt., 12 h, 69–86%; (e) R^1^SO_2_Cl, Et_3_N, DCM, rt., 12 h, 18–86%; (f) R^1^SO_2_NH_2_, Et_3_N, 2-chloro-1-methylpyridinium iodide, cat. DMAP, DCM, rt., 12 h, 6–88%; (g) *rac*-, (*R*)-, or (*S*)-**12**, DIPEA, ACN, reflux, 4 h, 80–84%; (h) methanesulfonyl chloride, Et_3_N, DCM, rt., 4 h, 92–97%; (i) morpholine, 80 °C, 3 h, 85–87%; (j) TFA, DCM, 16 h, 99%. ACN, acetonitrile; DCM, dichloromethane; DIPEA, *N*,*N*-diisopropylethylamine; DMAP, 4-dimethylaminopyridine; THF, tetrahydrofuran; TFA, trifluoroacetic acid.

Synthetic derivatives related to hit **1** containing different sulfonamide groups (compounds **2–6** and **S1-S64**, Tables 1 and S1) were obtained as indicated in Scheme 1. Thus, common intermediate **11** was synthesized from 4-trifluoromethylbenzonitrile via reaction with hydroxylamine to form an *N*′-hydroxy-4-arylcarboximidamide, followed by acylation with chloroacetyl chloride and in situ condensation to obtain the 1,2,4-oxadiazole ring. Next, reaction with the appropriated substituted piperidine provided intermediates **S88**–**S91**, which after deprotection or hydrolysis of the amino or ester group, respectively, and subsequent coupling with the corresponding sulfonyl derivative afforded sulfonamides **2**–**5** and **S1**–**S36**, and acylsulfonamides **6** and **S37**–**S64**. Identified commercial allosteric potentiators **7–10** (Table 1) selected for in vivo assays were prepared by reaction of intermediate **11** with the appropriate substituted piperidine (Scheme 1). 3-(Morpholin-4-ylmethyl)piperidine (**12**), necessary for the synthesis of final compound **9**, was obtained starting from *tert*-butyl 3-(hydroxymethyl)piperidine-1-carboxylate, which was transformed into mesylate derivative **13** for its reaction with morpholine and subsequent deprotection with trifluoroacetic acid (Scheme 1).

**Scheme 2:**
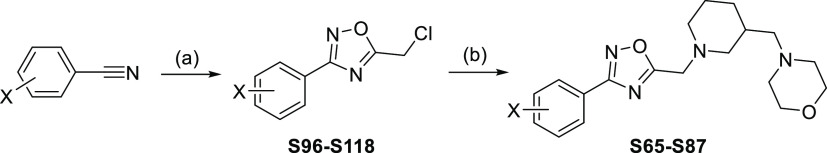
Synthesis of Compounds **S65-S87** Reagents and conditions: (a) (i) hydroxylamine hydrochloride, NaHCO_3_, ethanol, reflux, 16 h, 95–99%, (ii) chloroacetyl chloride, pyridine, 1,2-dichloroethane, reflux, 4 h, 25–56%; (b) *rac*-**12**, DIPEA, ACN, reflux, 4 h, 40–91%. ACN, acetonitrile; DIPEA, *N*,*N*-diisopropylethylamine.

New analogues of compound **9** bearing different substituents in the phenyl ring (compounds **S65**–**S87**, Table S3) were synthesized as shown in Scheme 2. The corresponding chloromethyloxadiazole intermediates **S96–S118** were prepared starting from the proper benzonitriles, according to the synthetic approach described for **11**; subsequent reaction with derivative **12** afforded target final compounds.

### In Vitro Characterization of Compound **9**

Based on the above-described results from the set of screening functional tests, compound **9** was selected for further pharmacological characterization at the GLP-1R.

#### cAMP Accumulation in hGLP-1R-Transfected Cells

**Figure 4:**
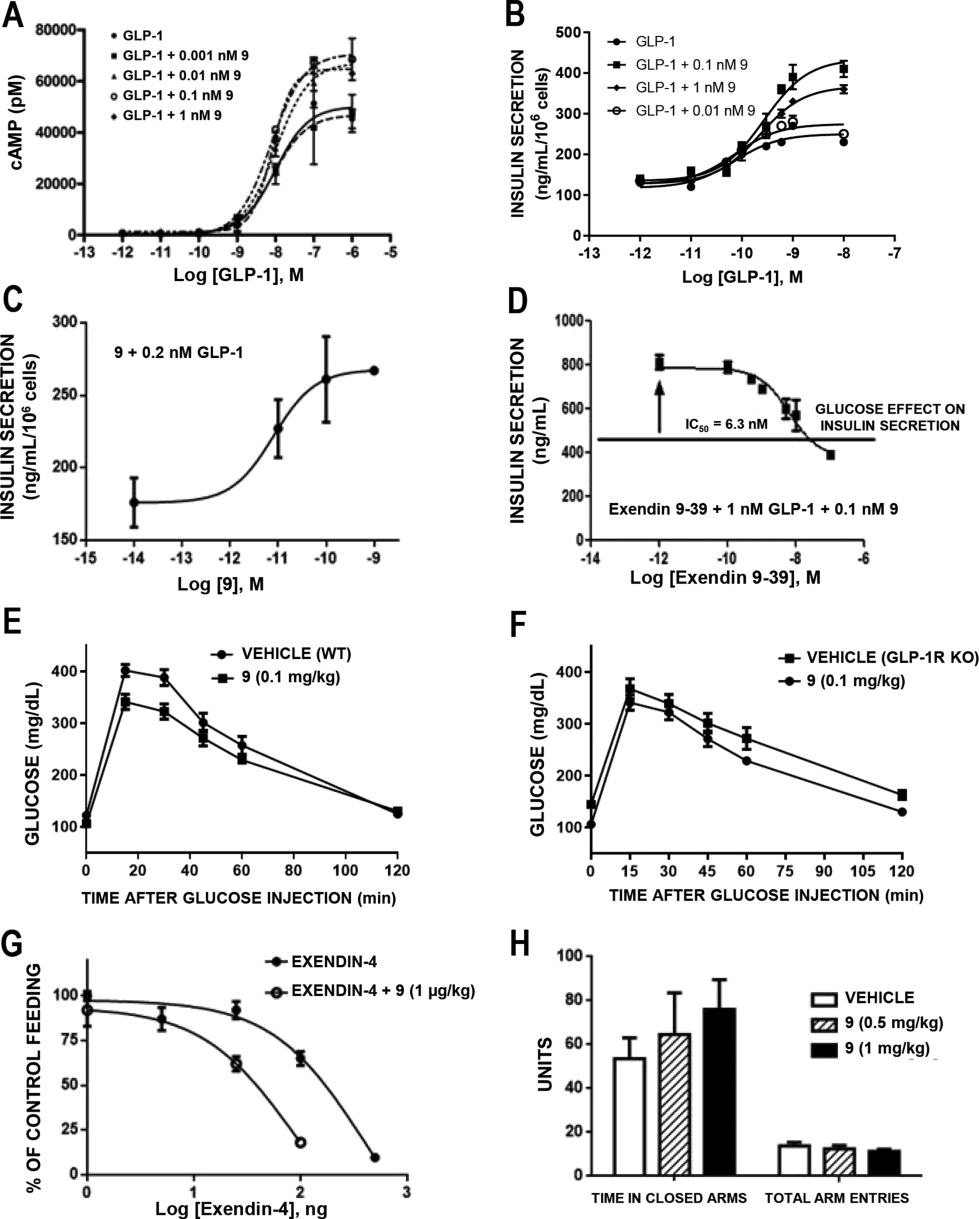
Characterization of compound **9**. (A) Potentiating effects of **9** on cAMP accumulation on HEK-GLP-1 cells stimulated by increasing doses of GLP-1. (B) Potentiation of the incretin effect of GLP-1 by a fixed concentration of **9** on INS-1E insulinoma cells. (C) Potentiation of the incretin effect of a fixed concentration of 0.2 nM GLP-1 by increasing concentrations of **9** on INS-1 insulinoma cells. An EC_50_ value of 0.008 nM was obtained (confidence interval, CI = 0.002–0.03 nM). (D) The GLP-1R antagonist exendin(9–39)-NH_2_ blocks the effects of **9** + GLP-1 on insulin release on INS-1E insulinoma cells. (E) **9** (0.1 mg/kg, ip) administered to wild-type C57BL/6N male mice improves glucose handling after an ip 2 g/kg glucose load. (F) No effect on glucose handling was seen for **9** (0.1 mg/kg, ip) administered to GLP-1R KO mice. (G) **9** (1 μg/kg injected in 5 μL, icv) potentiates exendin-4 (1, 5, 25, 200 and 500 ng injected in 5 μL, icv)-induced inhibition of feeding in male Wistar rats. Treatment with **9** lowered the IC_50_ of the feeding inhibitory effect of exendin-4 from a dose of 394 ng (CI = 172 to 903 ng) to 89 ng (CI = 15 to 509 ng). (H) **9** (0.5 and 1 mg/kg, doses effective for reducing time spent in hyperglycemia in glucose tolerance tests) does not induce anxiety-like behavior in male Wistar rats as measured in the elevated plus-maze. Points represent the mean ± SD (vertical bars) of triplicate measurements (in vitro) or 8 measures (in vivo).

cAMP production stimulated by increasing concentrations of GLP-1 was determined in HEK-293 cells expressing GLP-1R, in the presence of compound **9**. A clear concentration-dependent potentiation activity was observed for **9** in the range of 10^–12^ to 10^–9^ M, as shown in Figure 4A. Maximum efficacy was achieved at 0.1 nM concentration of compound **9** that produced a maximal potentiation effect of GLP-1R stimulation 42% higher than that of GLP-1, and it was demonstrated to be a reversible effect (Figure S1). Changes in EC_50_ value of GLP-1 were not significant (10.1 nM for GLP-1 alone versus 7.7 nM in the presence of 0.1 nM **9**) (Figure 4A). In addition, compound **9** was also able to potentiate cAMP production stimulated by GLP-1R agonist exendin-4 in a dose-dependent manner (Figure S2).

#### Insulin Release in Rat INS-1 Insulinoma Cells

Based on the potency observed in the cAMP assay, the effects of several concentrations of compound **9** (0.01, 0.1, and 1 nM) on insulin release induced by GLP-1 under high glucose conditions were studied in INS-1 β-cells (Figure 4B). The best response was observed at 0.1 nM of **9**, where a 1.8-fold potentiation of insulin secretion was detected, with an EC_50_ value of 0.25 nM. When a fixed concentration of 0.2 nM of GLP-1 was added to the dose–response curve of **9**, the maximal response observed reached a plateau at 0.1 nM, with an EC_50_ value of 0.008 nM (Figure 4C). These data confirm the extraordinary potency of this allosteric modulator. Importantly, the potentiation of insulin secretion promoted by GLP-1 in the presence of 0.1 nM **9** was antagonized by the GLP-1R antagonist exendin(9–39)-NH_2_ (IC_50_ = 6.3 nM, Figure 4D), which supports the effect of the compound is mediated by the GLP-1R.

#### Target Selectivity

Compound **9** was assayed for binding affinity in a panel of 54 GPCRs. At a concentration of 10 μM, a displacement lower than 50% of the specific binding was determined in all cases (Table S4), indicating that this compound is not acting at the orthosteric site of any of the tested receptors.

### In Vivo Characterization of Compound **9**

#### Glucose Handling

As previously observed in rats (Figure 3E), administration of compound **9** (0.1 mg/kg, ip) to wild-type C57BL/6N mice improved glucose handling after a parenteral (ip) glucose load (Figure 4E), whereas this effect was not observed in GLP-1R knockout (KO) mice (Figure 4F). Similar effects were observed when glucose was administered by the oral route (Figure S3). These data further support the selectivity of compound **9** for the GLP-1R. The improvement of glucose release was also observed after administration of **9** (2 but not 0.1 mg/kg, ip) in diabetic fatty Zucker rats, where the glucose handling improvement was characterized to occur along the first phase of insulin secretion promoted by the glucose load (2 g/kg), an effect typically mediated by incretin signals such as GLP-1 (Figure S4).

#### Effects on Feeding Inhibition

The injection of compound **9** (1 μg/kg, icv) was found to potentiate the feeding inhibition induced by the potent GLP-1R agonist exendin-4 (Figure 4G). Thus, while the IC_50_ of the feeding inhibitory effect of exendin-4 occurred at a dose of 394 ng, treatment with **9** lowered this IC_50_ to 89 ng. The in vitro potentiation of exendin-4 by compound **9** (see Figure S2) supports that the effect in feeding is mediated by the GLP-1R.

#### Effects on Anxiety-Like Behavior

Since activation of GLP-1R can induce anxiety and malaise, we tested the appearance of anxiety-like behaviors in Wistar rats after the administration of compound **9** (1 and 0.5 mg/kg, ip). Results showed that this PAM did not result in the emergence of anxiety-like behaviors nor hypolocomotion, as measured in the elevated plus-maze (Figure 4H).

### Pharmacokinetics of Compound **9**

Following with the preclinical development of **9**, pharmacokinetic studies were addressed. PAMPA-BBB data suggest a moderate brain penetration for compound **9** (Table S5). Clearance in a microsomal stability test yielded a half-life time greater than 30 min (Table S6), and low inhibitory effect was determined on P450 liver cytochrome activity (Table S7). In addition, **9** is a weak inhibitor of the hERG channel at micromolar concentrations (Table S8), far away from those needed to act as a GLP-1R allosteric modulator. In vivo pharmacokinetic experiments (Table 2, Figure S5) revealed a half-life time of around 3 h in plasma (2 mg/kg, iv and 10 mg/kg, po) and a quick absorption by the oral route, showing a maximal concentration at 50 min post-ingestion. The oral bioavailability of **9** was found to be 39%. Overall, the pharmacokinetic profile of compound **9** in rats can be considered promising for an orally active drug.

**Table 2:** In Vivo Pharmacokinetic Data of **9** in Rats after Oral and Intravenous Administration (Mean ± SD, *N* = 4)

route/dose (mg/kg)	*T*_max_ (h)	*C*_0_ (ng/mL)	*C*_max_ (ng/mL)	AUC_0–*t*_ (h*ng/mL)	AUC_0–inf_ (h*ng/mL)	*T*_1/2_ (h)	CL (mL/min/kg)	*V*_ss_ (L/kg)	*F* (%)
IV (2)	NA	491.99 ± 81.89	NA	277.61 ± 20.02	310.92 ± 12.94	3.25 ± 1.03	107.34 ± 4.58	18.05 ± 6.62	NA
PO (10)	0.83 ± 0.29	NA	184.91 ± 14.54	540.25 ± 132.15	644.45 ± 266.94	2.86 ± 1.88	NA	NA	38.92 ± 9.52

### Enantiomers of Compound **9**: Synthesis and Pharmacological Characterization

Since identified GLP-1R PAM **9** contains a stereocenter, the evaluation of the activity of each enantiomer is mandatory. Hence, an enantioselective synthesis was set up. According to the synthetic route used for the preparation of the racemic compound, (*R*)- and (*S*)-**9** were obtained by reaction of intermediate **11** with the appropriate enantiomer of piperidine **12** (see Scheme 1). (*R*)- and (*S*)-**12** were synthesized starting from the corresponding enantiomerically pure form of *tert*-butyl 3-(hydroxymethyl)piperidine-1-carboxylate.

**Figure 5:**
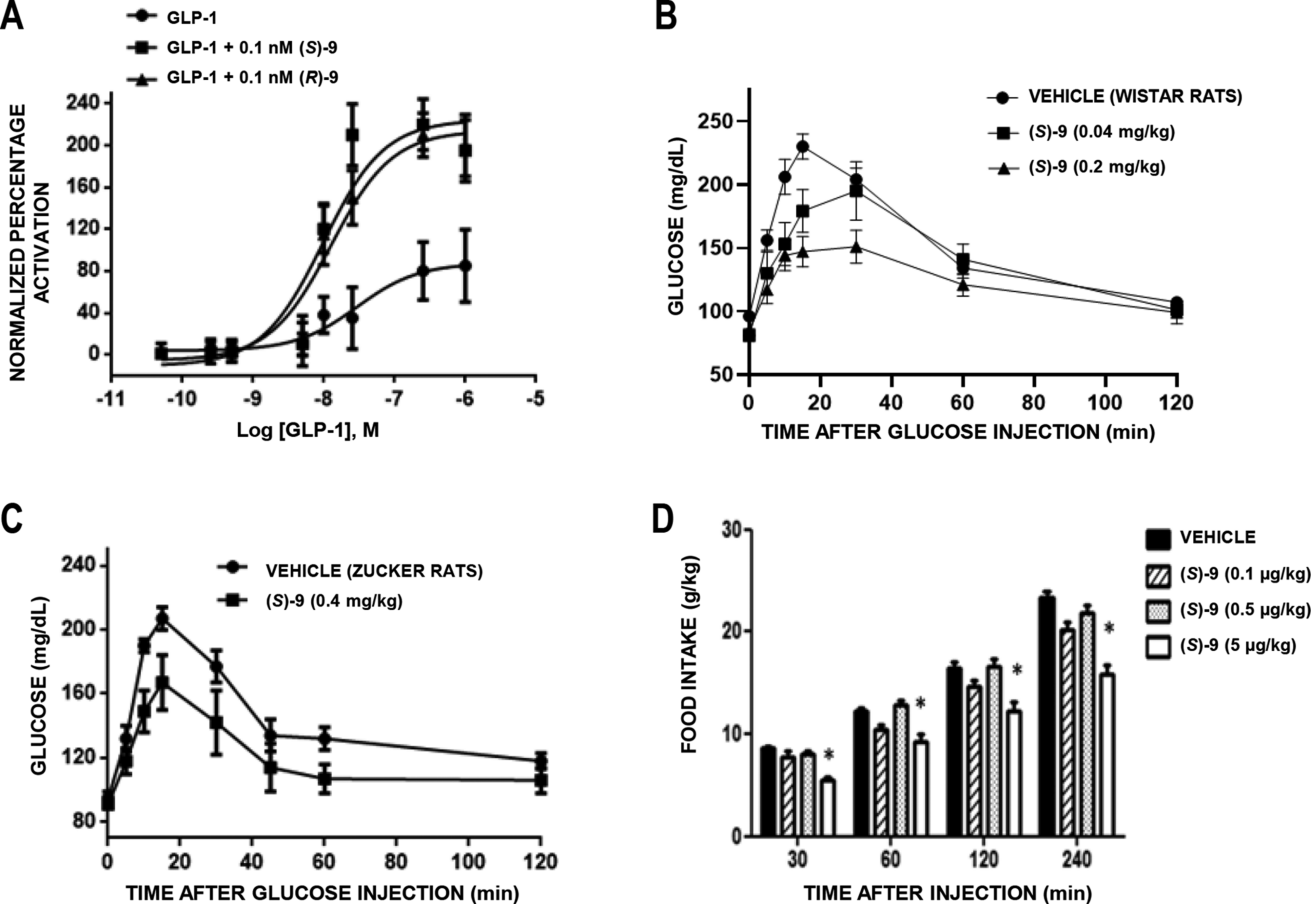
Characterization of (*S*)- and (*R*)-**9**. (A). Both enantiomers potentiate calcium fluxes in GLP-1R expressing cells with the same efficacy. (B) The (*S*) enantiomer (0.04 and 0.2 mg/kg, ip) improved glucose handling after ip injection of 2 g/kg glucose in 12-h fasted male Wistar rats. (C) The (*S*) enantiomer (0.4 mg/kg administered intragastrically) is orally active, improving glucose handling after ip injection of 2 g/kg glucose in 12-h fasted fatty Zucker rats. (D) Injection of (*S*)-**9** (0.1, 0.5, and 5 μg/kg in 5 μL, icv) reduced feeding in 12-h fasted male Wistar rats. Points represent the mean ± SD (vertical bars) of triplicate measurements (in vitro) and 8 measures (in vivo).

Data of the pharmacological characterization of (*R*)- and (*S*)-**9** are shown in Figures 5, S6, and S7. Both enantiomers (0.1 nM) were capable of activating calcium fluxes in HEK cells stably expressing hGLP-1R (Figure 5A) and induced a similar potentiation of the receptor activation stimulated by GLP-1, with a two-fold increase of the maximum agonist signal. In this in vitro assay, the same efficacy (EC_50_ = 10 nM) was also obtained for both (*S*)- and (*R*)-**9**, compared to 45 nM for GLP-1. The compounds were also able to similarly potentiate insulin secretion stimulated by GLP-1 in the stable human pancreatic cell line EndoC-βH1 under high glucose concentration (Figure S6A), whereas in rat INS-1E insulinoma cells the profile of both compounds under low glucose was similar to that observed for GLP-1; the moderate stimulation of insulin secretion observed for (*R*)- and (*S*)-**9** might be attributed to the release of GLP-1 from INS**-**1E cells (Figure S6B). According to the comparable in vitro behavior observed for both enantiomers, (*S*)-**9** was used for assessment of in vivo activity. Thus, this enantiomer (0.04 and 0.2 mg/kg, ip) clearly improved glucose handling after injection of 2 g/kg glucose in normal male Wistar rats (Figure 5B), was orally active (0.4 mg/kg) in fatty diabetic Zucker rats (Figure 5C), and was inactive in GLP-1R KO mice (Figure S7). In addition, (*S*)-**9** (5 μg/kg, icv) decreased feeding, and the effect lasted up to 2 h after injection (Figure 5D). Further studies of compounds (*S*)- and (*R*)-**9** will support their suitability as candidates for ulterior clinical development.

## Conclusions

In this work we have identified compound **9** (4-{[1-({3-[4-(trifluoromethyl)phenyl]-1,2,4-oxadiazol-5-yl}methyl)piperidin-3-yl]methyl}morpholine, V-0219) as an allosteric modulator of the GLP-1R that exhibits enhanced efficacy of GLP-1R stimulation dose-dependently, a subnanomolar potency in the potentiation of insulin secretion, and no observable off-target activities. The compound showed a remarkable in vivo activity, reducing food intake and improving glucose handling in normal and diabetic rodents, which was not observed in the genetic absence of the GLP-1R in a KO mouse model. The hERG and selectivity profiles were suitable for further development. Notably, synthesized (*S*)-**9** enantiomer was found to show oral efficacy in animal models. The characteristics of this compound are valuable as an orally active PAM of the GLP-1R. These results support the interest of this class of small-molecule drugs as a promising therapeutic approach for the increasingly prevalent obesity-associated diabetes.

## Experimental Section

### Synthesis

Unless otherwise stated, the starting materials, reagents, and solvents were purchased as high-grade commercial products from Sigma-Aldrich, Acros, or Scharlab, and were used without further purification. Triethylamine, *N*,*N*-diisopropylethylamine (DIPEA), and pyridine were dried over potassium hydroxide pellets, filtered, and distilled from calcium hydride. 1,2-Dichloroethane was distilled from calcium hydride. Dichloromethane (DCM) and diethyl ether were dried by passing the previously degassed solvents through activated alumina columns using a Pure Solv Micro 100 Liter solvent purification system. Analytical thin-layer chromatography (TLC) was run on Merck silica gel plates (Kieselgel 60F-254) with detection by UV light (254 nm), 5% ninhydrin solution in ethanol, or 10% phosphomolybdic acid solution in ethanol. Flash chromatography was performed on a Varian 971-FP flash purification system using silica gel cartridges (Varian, particle size 50 μm). Melting points (*M*_p_) were determined on a Stuart Scientific electrothermal apparatus. Infrared (IR) spectra were measured on a Bruker Tensor 27 instrument equipped with a Specac ATR accessory of 5200–650 cm^–1^ transmission range; frequencies (ν) are expressed in cm^–1^. Nuclear magnetic resonance (NMR) spectra were recorded on a Bruker Avance 500 MHz (^1^H, 500 MHz; ^13^C, 125 MHz) or Bruker DPX 300 MHz (^1^H, 300 MHz; ^13^C, 75 MHz) at room temperature (rt.) at the Universidad Complutense de Madrid (UCM) NMR facilities. Bruker DPX 300 MHz equipment was used unless otherwise stated. Chemical shifts (δ) are expressed in parts per million relative to the residual solvent peak for ^1^H and ^13^C nucleus (CDCl_3_: δ_H_ = 7.26, δ_C_ = 77.16; methanol-*d4*: δ_H_ = 3.31, δ_C_ = 49.00; DMSO-*d6*: δ_H_ = 2.50, δ_C_ = 39.52); coupling constants (*J*) are in hertz (Hz). The following abbreviations are used to describe peak patterns when appropriate: s (singlet), d (doublet), t (triplet), q (quartet), m (multiplet), and br (broad).

For all final compounds, purity was determined by high-performance liquid chromatography (HPLC) coupled to mass spectrometry (MS) using an Agilent 1200LC-MSD VL instrument, and satisfactory chromatograms confirmed a purity of at least 95% for all tested compounds. LC separation was achieved with an Eclipse XDB-C18 column (5 μm, 4.6 mm × 15 mm) together with a guard column (5 μm, 4.6 mm × 12.5 mm). The gradient mobile phases consisted of A (95:5 water:acetonitrile) and B (5:95 water:acetonitrile) with 0.1% ammonium hydroxide and 0.1% formic acid as solvent modifiers. MS analysis was performed with an electrospray ionization (ESI) source. Spectra were acquired in positive or negative ionization mode from 80 to 800 *m/z* and in UV-mode at four different wavelengths (210, 230, 254, and 280 nm). Elemental analyses (C, H, N) were obtained on a LECO CHNS-932 apparatus at the Elementary Chemical Analysis Laboratory of the Universidad Autónoma de Madrid, and were within 0.5% of the theoretical values, confirming a purity of at least 95% for all tested compounds.

Optical rotations were recorded on an Anton Paar MCP 100 modular circular polarimeter using a 1 dm path length, and concentrations are given as g/100 mL. The enantiomeric excess (ee) was determined by chiral HPLC analysis carried out on an Agilent 1200 series system using a Chiralpak IA column and 90/10 hexane/isopropanol mixture as mobile phase. Detection was performed with a diode array detector at 240 nm.

Racemic and enantiopure *tert*-butyl 3-{[(methylsulfonyl)oxy]methyl}piperidine-1-carboxylate (**13**)^29−31^ were synthesized according to previously reported procedures and spectroscopic data are in agreement with those reported.

### General Procedure for the Synthesis of Intermediates **11**, **S96–S118**

*N*′-hydroxy-4-arylcarboximidamide intermediates were synthesized according to previously described.^28^ Briefly, hydroxylamine hydrochloride (1.5 equiv) and NaHCO_3_ (2 equiv) were added to a solution of the corresponding benzonitrile (1 equiv, 2 mmol scale for intermediates **S96–S118**) in absolute ethanol (1.2 mL/mmol) at rt. under an argon atmosphere, and the reaction was refluxed for 15 h. Then the mixture was cooled to rt., filtered, and evaporated under reduced pressure. The residue was dissolved in ethyl acetate and washed with water and a saturated aqueous solution of NaCl. The organic layer was dried over Na_2_SO_4_, filtered, and evaporated under reduced pressure to yield the corresponding *N*′-hydroxy-4-arylcarboximidamide as a white solid (95–99% yield), which was used in the next step without further purification.

Pyridine (3 equiv) was added to a solution of the corresponding *N*′-hydroxy-4-arylcarboximidamide described above (1 equiv, 1.5 mmol scale) in anhydrous 1,2-dichloroethane (1 mL/mmol) at 0 °C under an argon atmosphere. A solution of chloroacetyl chloride (1.3 equiv) in anhydrous 1,2-dichloroethane (0.2 mL/mmol) was added dropwise, and the reaction mixture was then refluxed for 4 h. After cooling to rt., an aqueous 1.5 M solution of HCl was added till pH 1 and the organic layer was separated. The aqueous layer was extracted with ethyl acetate, and the combined organic layers were washed with water and a saturated aqueous solution of NaCl, dried (Na_2_SO_4_), filtered and evaporated under reduced pressure. The residue was purified by flash chromatography (hexane to 7:3 hexane/ethyl acetate) to yield the corresponding intermediate **11** or **S96–S118** (25–26% yield, Table S3).

### 5-(Chloromethyl)-3-[4-(trifluoromethyl)phenyl]-1,2,4-oxadiazole (**11**)

Following the previous general procedure, **11** was obtained from 4-(trifluoromethyl)benzonitrile (4.8 g, 28 mmol) in 48% yield (3.4 g) as a pale yellow oil. Chromatography: hexane to 7:3 hexane/ethyl acetate. Spectroscopic data are in agreement with those reported.^32^*R*_f_ = 0.75 (hexane/ethyl acetate, 1:1). ^1^H-NMR (CDCl_3_): δ 4.77 (s, 2H, CH_2_), 7.75 (d, *J* = 8.2, 2H, 2CH_Ar_), 8.21 (d, *J* = 8.2, 2H, 2CH_Ar_). ^13^C-NMR (CDCl_3_): δ 33.4 (CH_2_), 123.8 (q, *J* = 272.5, CF_3_), 126.1 (q, *J* = 3.7, 2CH_Ar_), 128.0 (2CH_Ar_), 129.7 (C_Ar_), 133.4 (q, *J* = 32.4, CCF_3_), 168.1 (C(N)=N), 175.0 (C(O)=N). MS (ESI): 261.1 (M – H)^−^.

### General Procedure for the Synthesis of Intermediates **S88–S91** and Compounds **7, 8,** and **10**

Sodium iodide (0.5 equiv), Cs_2_CO_3_ (2 equiv) and the corresponding piperidine derivative (1.1 equiv) were added to a solution of intermediate **11** (1 equiv) in anhydrous acetonitrile (5 mL/mmol) under an argon atmosphere, and the reaction mixture was refluxed until consumption of starting material (4–12 h). Then, the mixture was cooled to rt. and the solvent was evaporated under reduced pressure. The residue was dissolved in ethyl acetate and washed with a saturated aqueous solution of NaCl. The organic layer was dried (Na_2_SO_4_), filtered and evaporated under reduced pressure. The crude was triturated with hexane, filtered, and dried in the case of intermediates **S88–S91** (57–96% yield, Table S1), or purified by flash chromatography for final compounds **7**, **8**, and **10**.

The free amines of compounds **7**, **8**, and **10** were characterized (IR, NMR, HPLC-MS) and transformed into the corresponding hydrochloride salts. Thus, a 2 M HCl in Et_2_O solution (3 mL/mmol) was added to a solution of the free amine in anhydrous Et_2_O or DCM (6 mL/mmol) under an argon atmosphere, and the mixture was allowed to stand for 2 h. The hydrochloride salt was isolated by filtration or evaporation, washed with anhydrous diethyl ether and dried under high vacuum, to obtain an off-white solid that was characterized (HPLC-MS and elemental analysis).

#### 1-({3-[4-(Trifluoromethyl)phenyl]-1,2,4-oxadiazol-5-yl}methyl)piperidine-4-carboxamide (**7**)

Following the previous procedure, **7** was obtained from piperidine-4-carboxamide (38 mg, 0.30 mmol) and **11** (75 mg, 0.285 mmol) in 89% yield (90 mg). Chromatography: DCM to 9:1 DCM/MeOH. *M*_p_ = 187–188 °C. *R*_f_ = 0.38 (DCM/MeOH, 9:1). IR (ATR): ν 1628, 1428, 1326, 1123. ^1^H-NMR (DMSO-*d6*): δ 1.57 (ddd, *J* = 24.5, 12.1, 3.6, 2H, CH_2_), 1.69 (dd, *J* = 12.7, 2.7, 2H, CH_2_), 2.04 (tt, *J* = 11.4, 3.9, 1H, CH), 2.18 (td, *J* = 11.3, 2.4, 2H, CH_2_N), 2.92 (br d, *J* = 11.3, 2H, CH_2_N), 3.98 (s, 2H, CH_2_pip), 6.74 (s, 1H, NH), 7.21 (s, 1H, NH), 7.95 (d, *J* = 8.2, 2H, 2CH_Ar_), 8.23 (d, *J* = 8.1, 2H, 2CH_Ar_). ^13^C-NMR (DMSO-*d6*): δ 28.4 (2CH_2_), 41.1 (CH), 52.2 (2CH_2_N), 52.4 (CH_2_pip), 123.8 (q, *J* = 272.5, CF_3_), 126.3 (q, *J* = 3.8, 2CH_Ar_), 127.9 (2CH_Ar_), 130.0 (C_Ar_), 131.4 (q, *J* = 32.0, CCF_3_), 166.5 (C(N)=N), 176.3 (CONH_2_), 177.7 (C(O)=N). MS (ESI): 355.1 (M + H)^+^. HPLC-MS retention time: 13.29 min. Elemental analysis calculated for C_16_H_17_F_3_N_4_O_2_·HCl·H_2_O: C, 47.01; H, 4.93; N, 13.71; found: C, 47.32; H, 5.01; N, 13.98.

#### 3-Phenyl-1-[1-({3-[4-(trifluoromethyl)phenyl]-1,2,4-oxadiazol-5-yl}methyl)piperidin-3-yl]propan-1-one (**8**)

Following the previous procedure, **8** was obtained from 3-phenyl-1-piperidin-3-ylpropan-1-one (75 mg, 0.342 mmol) and **11** (75 mg, 0.285 mmol) in 84% yield (107 mg). Chromatography: hexane to 8:2 hexane/ethyl acetate. *R*_f_ = 0.7 (hexane/ethyl acetate, 1:1). IR (ATR): ν 3062, 1707, 1448, 1323, 1167, 1125. ^1^H-NMR (CDCl_3_): δ 1.35 (ddd, *J* = 24.4, 11.8, 4.4, 1H, 1/2CH_2pip_), 1.61–1.82 (m, 2H, CH_2pip_), 1.83–1.96 (m, 1H, 1/2CH_2pip_), 2.25 (td, *J* = 11.0, 3.1, 1H, 1/2CH_2_N_pip_), 2.39 (t, *J* = 10.6, 1H, 1/2CH_2_N_pip_), 2.68 (tt, *J* = 10.8, 3.5, 1H, CH), 2.75–2.83 (m, 2H, COCH_2_), 2.84–2.93 (m, 3H, 1/2CH_2_N_pip_, CH_2_Ph), 3.02 (br d, *J* = 11.2, 1H, 1/2CH_2_N_pip_), 3.92 (s, 2H, CH_2_pip), 7.13–7.21 (m, 3H, 3CH_Ph_), 7.26 (t, *J* = 7.1, 2H, 2CH_Ph_), 7.74 (d, *J* = 8.3, 2H, 2CH_Ar_), 8.22 (d, *J* = 8.2, 2H, 2CH_Ar_). ^13^C-NMR (CDCl_3_): δ 24.7 (CH_2pip_), 26.2 (CH_2pip_), 29.7 (CH_2_Ph), 42.8 (COCH_2_), 49.2 (CH), 53.6 (CH_2_pip, CH_2_N_pip_), 54.8 (CH_2_N_pip_), 123.9 (q, *J* = 271.0, CF_3_), 126.0 (q, *J* = 3.7, 2CH_Ar_), 126.3 (CH_Ph_), 128.0 (2CH_Ar_), 128.4 (2CH_Ph_), 128.6 (2CH_Ph_), 130.1 (C_Ar_), 133.1 (q, *J* = 32.6, CCF_3_), 141.2 (C_Ph_), 167.5 (C(N)=N), 176.9 (C(O)=N), 210.8 (C=O). MS (ESI): 443.9 (M + H)^+^. HPLC-MS retention time: 24.71 min. Elemental analysis calculated for C_24_H_24_F_3_N_3_O_2_·HCl·0.5H_2_O: C, 58.96; H, 5.36; N, 8.59; found: C, 59.27; H, 5.23; N, 8.56.

#### 2-{2-[1-({3-[4-(Trifluoromethyl)phenyl]-1,2,4-oxadiazol-5-yl}methyl)piperidin-2-yl]ethyl}pyridine (**10**)

Following the previous procedure, **10** was obtained from 2-(2-piperidin-2-ylethyl)pyridine (56 μL, 0.29 mmol) and **11** (72 mg, 0.274 mmol) in 84% yield (96 mg). Chromatography: hexane/ethyl acetate, 10:1 to 1:1. *R*_f_ = 0.12 (hexane:ethyl acetate, 1:1). IR (ATR): ν 2934, 2856, 1592, 1420, 1324, 1169, 1128. ^1^H-NMR (CDCl_3_): δ 1.25–1.33 (m, 1H, 1/2CH_2pip_), 1.43–1.64 (m, 3H, 1/2CH_2pip_, CH_2pip_), 1.71–1.81 (m, 2H, CH_2pip_), 2.00–2.16 (m, 2H, CH_2_CH_2_Py), 2.44–2.54 (m, 2H, CH, 1/2CH_2_N_pip_), 2.78–3.02 (m, 3H, 1/2CH_2_N_pip_, CH_2_Py), 4.14 (d, *J* = 13.9, 2H, CH_2_pip), 7.08 (ddd, *J* = 7.4, 4.9, 0.9, 1H, CH_Py_), 7.17 (d, *J* = 7.8, 1H, CH_Py_), 7.56 (td, *J* = 7.7, 1.9, 1H, CH_Py_), 7.74 (d, *J* = 8.1, 2H, 2CH_Ar_), 8.21 (d, *J* = 8.1, 2H, 2CH_Ar_), 8.51 (dd, *J* = 4.9, 0.9, 1H, CH_Py_). ^13^C-NMR (CDCl_3_): δ 23.8 (CH_2pip_), 25.5 (CH_2pip_), 30.3 (CH_2pip_), 32.1 (CH_2_CH_2_Py), 33.7 (CH_2_Py), 48.6 (CH_2_pip), 53.3 (CH_2_N_pip_), 59.3 (CH), 121.2 (CH_Py_), 122.9 (CH_Py_), 123.9 (q, *J* = 272.7, CF_3_), 125.9 (q, *J* = 3.6, 2CH_Ar_), 128.0 (2CH_Ar_), 130.4 (C_Ar_), 133.2 (q, *J* = 32.2, CCF_3_), 136.6 (CH_Py_), 149.4 (CH_Py_), 162.1 (C_Py_), 167.3 (C(N)=N), 177.8 (C(O)=N). MS (ESI): 417.2 (M + H)^+^. HPLC-MS retention time: 25.70 min. Elemental analysis calculated for C_22_H_23_F_3_N_4_O·2HCl·2H_2_O: C, 50.29; H, 5.56; N, 10.66; found: C, 50.41; H, 5.29; N, 10.60.

### General Procedure for the Synthesis of Intermediates **S92–S93**

A 4 M solution of HCl in dioxane (2.5 mL/mmol) was added to a solution of the corresponding *N*-Boc-protected intermediate **S88** or **S89** in anhydrous dioxane (2.5 mL/mmol) under an argon atmosphere, and the reaction was stirred at 50 °C for 12 h. After this time, the mixture was cooled to rt. and evaporated under reduced pressure. The residue was triturated with diethyl ether to yield the hydrochloride salt of the deprotected compound **S92** or **S93** as an off-white solid (71–86% yield, Table S1).

### General Procedure for the Synthesis of Intermediates **S94–S95**

To a stirred solution of ester derivative **S90** or **S91** (1 equiv) in a 1:1:0.2 mixture of THF/ethanol/water (8 mL/mmol) at 0 °C, LiOH·H_2_O (2 equiv) was added and the reaction was stirred at rt. for 12 h. Next, the mixture was concentrated under reduced pressure and the residue was dissolved in water and acidified until pH 2 using 1.5 N aqueous HCl. The resulting solid was filtered, washed with water, and dried under vacuum to afford **S94** or **S95** as an off-white solid (69–86% yield, Table S1).

### General Procedure for the Synthesis of Sulfonamide Derivatives **2**–**5**, **S1–S36**

Triethylamine (3.0 equiv) and the corresponding sulfonyl chloride derivative (1.1 equiv) were added to an ice-cooled solution of the hydrochloride salt of the appropriate intermediate **S92** or **S93** (1.0 equiv, 0.241–0.519 mmol scale) in anhydrous DCM (10 mL/mmol) under an argon atmosphere and the reaction was stirred at rt. for about 12 h. After completion of the reaction (monitored by TLC), the mixture was washed with 10% citric acid solution (5 mL) and brine (5 mL), and the organic phase was dried over Na_2_SO_4_ and evaporated under reduced pressure. The residue was recrystallized from ethanol to obtain the desired final product **2–5, S1**–**S36** as a solid (18–86% yield, Table S1).

### General Procedure for the Synthesis of Acylsulfonamide Derivatives **6**, **S37–S64**

The corresponding sulfonamide derivative (1.5 equiv) was added to an ice-cooled solution of the appropriate carboxylic acid intermediate **S94–S95** (1.0 equiv, 0.270 mmol scale) in anhydrous DCM (15 mL/mmol). Next, triethylamine (3.0 equiv), Mukaiyama reagent (2-chloro-1-methylpyridinium iodide, 1.2 equiv), and DMAP (0.2 equiv) were sequentially added, and reaction mixture was stirred at rt. for about 12 h. After completion of the reaction (monitored by TLC), the reaction mixture was washed with 10% citric acid solution (5 mL) and brine (5 mL), and the organic phase was dried over Na_2_SO_4_ and evaporated under reduced pressure. The residue was recrystallized from ethanol to obtain the desired final product **6**, **S37–S64** as a solid (6–88% yield, Table S1).

### General Procedure for the Synthesis of *rac*-, (*R*)-, (*S*)-**14**

A solution of racemic or enantiopure **13** and morpholine (0.8 mL/mmol) was heated at 80 °C for 3 h under an argon atmosphere. The reaction mixture was evaporated, and the residue was dissolved with ethyl acetate. The organic solution was washed with water and a saturated aqueous solution of NaCl, dried (Na_2_SO_4_), filtered and evaporated under reduced pressure. The residue was purified by flash chromatography (hexane to 6:4 hexane/ethyl acetate) to yield racemic **14** or the corresponding enantiomer as a white solid.

#### tert-Butyl 3-(morpholin-4-ylmethyl)piperidine-1-carboxylate (**14**)

Following the previous procedure, racemic **14** was obtained from racemic **13** (390 mg, 1.33 mmol) in 85% yield (322 mg). *M*_p_ = 58–59 °C. *R*_f_ = 0.31 (hexane/ethyl acetate, 1:1). IR (ATR): ν 2975, 2936, 1689, 1365, 1271, 1167, 1125. ^1^H-NMR (CDCl_3_): δ 1.06–1.13 (m, 1H, 1/2CH_2pip_), 1.48 (s, 10H, 3CH_3_, 1/2CH_2pip_), 1.58–1.82 (m, 3H, CH_2pip_, CH), 2.17–2.20 (m, 2H, CH_2_morph), 2.32–2.50 (m, 5H, 1/2CH_2_N_pip_, 2CH_2_N_morph_), 2.77 (br t, *J* = 11.2 , 1H, 1/2CH_2_N_pip_), 3.71–3.74 (m, 4H, 2CH_2_O_morph_), 3.94 (br d, *J* = 13.2, 1H, 1/2CH_2_N_pip_), 4.14 (br s, 1H, 1/2CH_2_N_pip_). ^13^C-NMR (CDCl_3_): δ 24.9 (CH_2pip_), 28.6 (3CH_3_), 29.6 (CH_2pip_), 33.2 (CH), 45.0 (CH_2_N_pip_), 52.2 (CH_2_N_pip_), 54.2 (2CH_2_N_morph_), 62.4 (CH_2_morph), 67.2 (2CH_2_O_morph_), 79.3 (C(CH_3_)_3_), 155.0 (CO). MS (ESI): 285.2 (M + H)^+^.

#### tert-Butyl (3*S*)-(-)-3-(morpholin-4-ylmethyl)piperidine-1-carboxylate [(*S*)-(-)-**14**]

Following the previous procedure, (*S*)-(-)-**14** was obtained from (*R*)-(-)-**13** (1.10 g, 3.75 mmol) in 85% yield (906 mg). [α]_D_^20^ = −6.3 (*c* = 1, CHCl_3_). Spectroscopic data were in agreement with those described for racemic **14**.

#### tert-Butyl (3*R*)-(+)-3-(morpholin-4-ylmethyl)piperidine-1-carboxylate [(*R*)-(+)-**14**]

Following the previous procedure, (*R*)-(+)-**14** was obtained from (*S*)-(+)-**13** (1.28 g, 4.38 mmol) in 87% yield (1.09 g). [α]_D_^20^ = +6.2 (*c* = 1, CHCl_3_). Spectroscopic data were in agreement with those described for racemic **14**.

#### General Procedure for the Synthesis of *rac*-, (*R*)-, (*S*)-**12**

Trifluoroacetic acid (TFA) (20 equiv) was added to a solution of racemic or enantiopure **14** (1 equiv) in anhydrous DCM (20 mL/mmol) at rt. under an argon atmosphere. The reaction mixture was stirred at rt. for 16 h and solvent was then removed under reduced pressure. The excess of TFA was removed by azeotropic distillation with toluene (2×) and the resulting residue was dried under vacuum to afford the trifluoroacetate salt of racemic **12** or of the corresponding enantiomer as a white solid.

#### 3-(Morpholin-4-ylmethyl)piperidinium trifluoroacetate (**12**)

Following the previous procedure, racemic **12** was obtained from racemic **14** (310 mg, 1.09 mmol) in 99% yield (447 mg). *M*_p_ = 70–72 °C. *R*_f_ = 0.15 (hexane/ethyl acetate, 1:1). IR (ATR): ν 3431, 1670, 1455, 1423, 1200, 1171, 1130. ^1^H-NMR (methanol-*d4*): δ 1.34–1.46 (m, 1H, 1/2CH_2pip_), 1.78–1.87 (m, 1H, 1/2CH_2pip_), 1.94–2.03 (m, 2H, CH_2pip_), 2.36–2.43 (m, 1H, CH), 2.80 (t, *J* = 12.2, 1H, 1/2CH_2_N_pip_), 2.93 (td, *J* = 12.7, 3.2, 1H, 1/2CH_2_N_pip_), 3.11–3.19 (m, 2H, CH_2_morph), 3.21–3.40 (br m, 4H, 2CH_2_N_morph_), 3.37 (br d, *J* = 12.6, 1H, 1/2CH_2_N_pip_), 3.53 (d, *J* = 12.5, 1H, 1/2CH_2_N_pip_), 3.93 (br s, 4H, 2CH_2_O_morph_). ^13^C-NMR (methanol-*d4*): δ 22.7 (CH_2pip_), 27.7 (CH_2pip_), 30.2 (CH), 44.9 (CH_2_N_pip_), 47.3 (CH_2_N_pip_), 53.7 (2CH_2_N_morph_), 60.7 (CH_2_morph), 64.8 (2CH_2_O_morph_). MS (ESI): 185.2 (M + H)^+^.

#### (3*R*)-(+)-3-(Morpholin-4-ylmethyl)piperidinium trifluoroacetate [(*R*)-(+)-**12**]

Following the previous procedure, (*R*)-(+)-**12** was obtained from (*S*)-(−)-**14** (890 mg, 3.13 mmol) in 99% yield (1.28 g). [α]_D_^20^ = +1.8 (*c* = 1, MeOH). Spectroscopic data were in agreement with those described for racemic **12**.

#### (3*S*)-(−)-3-(Morpholin-4-ylmethyl)piperidinium trifluoroacetate [(*S*)-(−)-**12**]

Following the previous procedure, (*S*)-(−)-**12** was obtained from (*R*)-(+)-**14** (1 g, 3.52 mmol) in 99% yield (1.43 g). [α]_D_^20^ = −2.4 (*c* = 1, MeOH). Spectroscopic data were in agreement with those described for racemic **12**.

#### General Procedure for the Synthesis of *rac*-, (*R*)-, (*S*)-**9**

DIPEA (6 equiv) and racemic or enantiopure **12** (1.1 equiv) were added to a solution of **11** (1 equiv) in dry acetonitrile (10 mL/mmol) under an argon atmosphere, and the reaction mixture was refluxed for 4 h. After this time, the reaction was cooled to rt. and evaporated under reduced pressure. The residue was dissolved in ethyl acetate and washed with a saturated aqueous solution of NaCl. The organic layer was dried (Na_2_SO_4_), filtered and evaporated under reduced pressure. The residue was purified by flash chromatography (DCM to DCM:methanol, 9:1) to yield racemic **9** or the corresponding enantiomer as a pale yellow syrup. The free amines of racemic and enantiopure forms of **9** were transformed into the corresponding hydrochloride salts as detailed above for compounds **7**, **8**, **10**.

#### 4-{[1-({3-[4-(Trifluoromethyl)phenyl]-1,2,4-oxadiazol-5-yl}methyl)piperidin-3-yl]methyl}morpholine (**9**)

Following the previous procedure, racemic **9** was obtained from **11** (202 mg, 0.769 mmol) and racemic **12** (349 mg, 0.846 mmol) in 80% yield (254 mg). *M*_p_ = 206–207 °C. *R*_f_ = 0.48 (DCM:methanol, 10:1). IR (ATR): ν 2934, 2854, 1322, 1167, 1122. ^1^H-NMR (CDCl_3_): δ 0.84–0.97 (m, 1H, 1/2CH_2pip_), 1.61–1.78 (m, 3H, 1/2CH_2pip_, CH_2pip_), 1.88–1.97 (m, 2H, 1/2CH_2_N_pip_, CH), 2.13–2.17 (m, 2H, CH_2_morph), 2.21 (td, *J* = 10.9, 3.2, 1H, 1/2CH_2_N_pip_), 2.31–2.52 (m, 4H, 2CH_2_N_morph_), 2.93 (br d, *J* = 10.7, 1H, 1/2CH_2_N_pip_), 3.09 (d, *J* = 7.2, 1H, 1/2CH_2_N_pip_), 3.63–3.71 (m, 4H, 2CH_2_O_morph_), 3.93 (d, *J* = 6.2, 2H, CH_2_pip), 7.74 (d, *J* = 8.2, 2H, 2CH_Ar_), 8.23 (d, *J* = 8.2, 2H, 2CH_Ar_). ^13^C-NMR (CDCl_3_): δ 25.1 (CH_2pip_), 28.9 (CH_2pip_), 33.4 (CH), 53.7 (CH_2_pip), 54.2 (CH_2_N_pip_, 2CH_2_N_morph_), 58.7 (CH_2_N_pip_), 63.1 (CH_2_morph), 67.1 (2CH_2_O_morph_), 123.9 (q, *J* = 270.8, CF_3_), 125.9 (q, *J* = 3.7, 2CH_Ar_), 127.9 (2CH_Ar_), 130.3 (C_Ar_), 133.1 (q, *J* = 32.4, CCF_3_), 167.4 (C(N)=N), 177.3 (C(O)=N). MS (ESI): 411.2 (M + H)^+^. HPLC-MS retention time: 18.88 min. Elemental analysis calculated for C_20_H_25_F_3_N_4_O_2_·2HCl·2H_2_O: C, 46.25; H, 6.02; N, 10.79; found: C, 46.47; H, 5.99; N, 10.91.

#### (+)-4-{[(3*S*)-1-({3-[4-(Trifluoromethyl)phenyl]-1,2,4-oxadiazol-5-yl}methyl)piperidin-3-yl]methyl}morpholine [(*S*)-(+)-**9**]

Following the previous procedure, (*S*)-(+)-**9** was obtained from **11** (202 mg, 0.769 mmol) and (*R*)-(+)-**12** (349 mg, 0.846 mmol) in 84% yield (265 mg). [α]_D_^20^ = +25.4 (*c* = 1, CHCl_3_). Enantiomeric excess >98% (Chiral HPLC retention time: 5.66 min). Elemental analysis calculated for C_20_H_25_F_3_N_4_O_2_·2HCl·3H_2_O: C, 44.70; H, 6.19; N, 10.43; found: C, 45.02; H, 6.02; N, 10.58. Spectroscopic data were in agreement with those described for racemic **9**.

#### (−)-4-{[(3*R*)-1-({3-[4-(Trifluoromethyl)phenyl]-1,2,4-oxadiazol-5-yl}methyl)piperidin-3-yl]methyl}morpholine [(*R*)-(−)-**9**]

Following the previous procedure, (*R*)-(−)-**9** was obtained from **11** (300 mg, 1.14 mmol) and (*S*)-(−)-**12** (518 mg, 1.26 mmol) in 83% yield (390 mg). [α]_D_^20^ = −25.9 (*c* = 1, CHCl_3_). Enantiomeric excess = 99% (Chiral HPLC retention time: 6.38 min). Elemental analysis calculated for C_20_H_25_F_3_N_4_O_2_·2HCl·2H_2_O: C, 46.25; H, 6.02; N, 10.79; found: C, 46.37; H, 5.86; N, 10.81. Spectroscopic data were in agreement with those described for racemic **9**.

### General Procedure for the Synthesis of Compounds **S65–S87**

To a solution of the appropriate intermediate **S96-S118** (1 equiv, 0.250 mmol scale) in dry acetonitrile (10 mL/mmol) under an argon atmosphere, DIPEA (6 equiv) and racemic **12** (1.1 equiv) were added and the reaction mixture was refluxed for 4 h. After this time, the reaction was cooled to rt. and evaporated under reduced pressure. The residue was dissolved in ethyl acetate and washed with a saturated aqueous solution of NaCl. The organic layer was dried over Na_2_SO_4_, filtered and evaporated under reduced pressure. The residue was purified by flash chromatography (DCM to DCM:methanol, 9:1) to yield the corresponding final compound **S65–S87** (Table S3). The free amines of the compounds were transformed into the corresponding hydrochloride salts as detailed for compounds **7–10**.

### Functional Activity at GLP-1R in Cell Lines Expressing hGLP-1R

HEK-GLP-1 cells were maintained in Dulbecco’s Modified Eagle Medium (DMEM) containing 10% FBS in the presence of 0.25 mg/mL hygromycin and 1 μg/mL puromycin at 37 °C in a 5% CO_2_ atmosphere. Cells were seeded in 96-well plates (10^4^ cells/well) in 20 μL Opti-MEM and grown for 24 h. After this time the culture medium was replaced by 20 μL fresh Opti-MEM containing 500 μM IBMX and 100 μM K579 and incubated with gentle stirring for 10 min at rt. Test compounds were added to the cells and incubated for 15 min at rt. After this time GLP-1(7-36)-NH_2_ or exendin-4 was added to the cells and incubated for 15 min at rt. cAMP formation was measured by employing the cAMP dynamic range kit from CisBio following manufacturer instructions. Concentration-response curves were fitted to a 4-parameter logistic equation by using GraphPad Prism 2.1 software.

### In Vitro Secretion of Insulin by Rat INS-1 and Human EndoC-βH1 Cells

Both cell lines were cultured in RPMI 1640 medium supplemented with 10% fetal calf serum as described. Cell monolayers were incubated at 37 °C in a 7.5% CO_2_ atmosphere. For insulin secretion studies, cells were detached, washed once with glucose-free medium and placed in 48 multiwell plates (100,000 cells per well) and kept in glucose-free medium for 1.5 h prior to incubation with drugs. The incubation was done using two types of culture media, defined as low glucose (0.5–3 mM) and high glucose (15 mM). Both conditions were selected for analyzing the glucose dependency of GLP-1(7-36)-NH_2_ actions, since this incretin acts at high glucose concentration. Compounds were tested both, alone and in association with GLP-1(7-36)-NH_2_ for excluding potential agonistic effects at the GLP-1R. Incubation with compounds lasted 30 min, and then the media was collected, centrifuged at 4 °C for 2 min until assayed for insulin using commercial ELISA kit.

### In Vivo Experiments

All procedures for animal experiments were conducted in adherence to the European Communities Council Directive (86/609/ECC) and Spanish regulations (BOE 252/34367-91, 2005) for the use of laboratory animals.

### Glucose Handling in Experimental Animals

Glucose tolerance test was carried out both in Wistar and Zucker (fa/fa) obese male rats and in wild-type and GLP-1R KO mice, by injecting an ip glucose load of 2 g/kg body weight (diluted in saline) or by oral administration of a glucose solution (2 g/kg in a volume of water of 1 mL/kg body weight). Animals were food-deprived overnight (12 h fasting) before testing. Thirty minutes before glucose load, animals received by ip administration the compound being tested dissolved in saline/DMSO (0.2%) as vehicle. Tail blood samples were collected before (0 min) and 5, 10, 15, 30, 45, 60, and 120 min after glucose administration. Glucose was determined using a commercial glucometer (Accu-check, Roche Diagnostic).

### Feeding Inhibition in Experimental Rodents

Stimulation of central GLP-1R induces satiety in food-deprived animals.^33,34^ Thus, we used icv administration of a GLP-1R agonist (exendin-4) for compound screening, testing satiety induced by either the compound alone or combined with a submaximal active dose of exendin-4 (100 ng, icv). Male Wistar rats (Panlab, Barcelona, Spain) weighing 400 ± 35 g at the start of the experiment were housed individually and maintained in a temperature and light-controlled environment on a 12-h light/dark cycle (lights on at 8:00 am) with free access to food and water. Zucker obese (fa/fa) animals and lean controls (fa/−) aged 12–16 weeks (Panlab, Barcelona, Spain) and weighing 380–450 g and 294–349 g, respectively, at the start of the experiments were used in the studies for peripheral and oral administration. For the icv administration of the different compounds, a 7-mm stainless steel guide cannula aimed at the lateral ventricle was implanted in the rats (the implantation coordinates were 0.6 mm posterior to the bregma, ±2.0 mm lateral, and 3.2 mm below the surface of the skull). After a 7-day postsurgical recovery period, cannula patency was confirmed by gravity flow of isotonic saline through an 8-mm-long 30-gauge injector inserted within the guide to 1 mm beyond its tip. This procedure allowed the animals to become familiar with the injection technique.

Animals were food-deprived overnight for 12 h prior to compound testing, and total amount of food and water intake was measured at 30, 60, 120, and 240 min post injection. Results were analyzed and presented either as g (food)/kg (body weight) or normalized as % over control group intake. Both GLP-1(7-36)-NH_2_ and exendin-4 were used as GLP-1R agonists and icv injected together with testing compounds or vehicle (0.2% DMSO in saline) in a volume of 5 μL. When oral administration was addressed, compounds were delivered intragastrically in a volume of 1 mL/kg.

### Anxiety-like Behavior^33^

Animals naïve to the maze were manipulated for 7 days before testing. The day of the experiment, 30 min after ip injection of **9**, animals were placed in the center of the maze, facing an open arm. The number of entries and the % of time spent in the exposed arms of the maze were measured. Test length was 5 min and animals were not retested.

## Supporting Information Available

Structures of all compounds (Tables S1–S3), CEREP panel results (Table S4), pharmacokinetic profile of compound **9** (detailed experimental procedures, Tables S5–S8, Figure S5), pharmacological characterization of compounds *rac*-, (*S*)-, and (*R*)-**9** (Figures S1–S4, S6, S7) (PDF)
Molecular formula strings (CSV)

## Abbreviations

br: broad
DIPEA: *N,N*-diisopropylethylamine
DMEM: Dulbecco’s modified Eagle medium
DPP-4: dipeptidyl peptidase
GLP-1R: glucagon-like peptide-1 receptor
KO: knockout
PAM: positive allosteric modulator
