# Supplementary material for: Discovery of V-0219: A Small-Molecule Positive Allosteric Modulator of the Glucagon-Like Peptide-1 Receptor toward Oral Treatment for “Diabesity”
Source: J Med Chem. 2022 Mar 29;65(7):5449–61. doi: 10.1021/acs.jmedchem.1c01842 (PMC9014410; doi:10.1021/acs.jmedchem.1c01842)
Supplement: Supplementary file 1 — jm1c01842_si_001.pdf [file jm1c01842_si_001.pdf]

Supporting Information

Discovery of V-0219: A Small-Molecule Positive Allosteric Modulator of the Glucagon-Like Peptide-1 Receptor toward Oral Treatment for "Diabesity"

Juan M. Decara,[Δ,a] *Henar Vázquez-Villa,* [Δ,b] *José Brea,*[c] *Mónica Alonso,*[a] *Raj Kamal Srivastava,*[#,d] *Laura Orio,*[e] *Francisco Alén,*[e] *Juan Suárez,*[a] *Elena Baixeras,*[a] *Javier García-Cárceles,*[b] *Andrea Escobar-Peña,*[b] *Beat Lutz,*[d] *Ramón Rodríguez,*[f] *Eva Codesido,*[f] *F. Javier Garcia-Ladona,*[g] *Teresa A. Bennett,*[§,h] *Juan A. Ballesteros,*[h] *Jacobo Cruces,*[f] *María I. Loza,*[c] *Bellinda Benhamú,*[b] *Fernando Rodríguez de Fonseca,*[*,a],[e] *and María L. López-Rodríguez*[*,b]

[a] Unidad de Gestión Clínica de Salud Mental, Instituto IBIMA, Hospital Regional Universitario, E-29010 Málaga, Spain. [b] Departamento de Química Orgánica, Universidad Complutense de Madrid, E-28040 Madrid, Spain. [c] Biofarma Research group. USEF Screening Platform, CIMUS, USC, E-15782 Santiago de Compostela, Spain. [d] Institute of Physiological Chemistry, University Medical Center of the Johannes Gutenberg, University of Mainz, 55128 Mainz, Germany. [e] Departamento de Psicobiología. Facultad de Psicología, Universidad Complutense de Madrid, E-28040 Madrid, Spain. [f] Galchimia, E-15823 O Pino, A Coruña, Spain. [g] ABAXYS Therapeutics, Rue du Berceau, 91, 1495 Villers-la-Ville, Belgium. [h] ViviaBiotech S.L., Parque Científico de Madrid, E-28760 Madrid, Spain.

[*] Corresponding authors: mluzlr@ucm.es, fernando.rodriguez@ibima.eu

Note

The compounds, synthetic pathways and data reported in the present article have been originated at Vivia Biotech S.L., Universidad Complutense, IBIMA, CIMUS, Galchimia, and University of Mainz. At present, they are property of ABAXYS Therapeutics S.A. (Belgium). Compounds have been protected and thus any use is strictly prohibited unless a license is agreed by the owner. Any request related to the compounds reported here should be addressed to F. J. Garcia-Ladona at ABAXYS Therapeutics S.A., FJGarciaL@abaxysth.com.

Table of Contents

1. Structure and synthetic data of compounds **2-6**, **S1**-**S87**, intermediates **S88-S118**, and commercial compounds S2
2. NMR spectra and HPLC trace analysis of compounds **7-10** S12
3. Binding to off-target receptors S23
4. Pharmacokinetics of compound **9** S25
5. Figures S30

1. Structure and synthetic data of compounds **2-6**, **S1**-**S87**, intermediates **S88-S118**, and commercial compounds

Table S1. Structure and synthetic data of compounds **2-6**, **S1-S64**, and intermediates **S88-S95**.

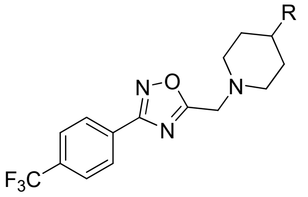

S1-S64, 2-6

Compound	R	Quantity	Yield (%)	Purity (%, HPLC-MS)	MS (ESI, $[M+H]^+$)
**S1**	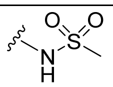	29 mg	29	99	405.1
**S2**	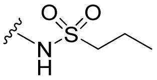	31 mg	28	99	433.1
**S3**	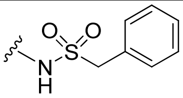	22 mg	18	99	481.1
**S4**	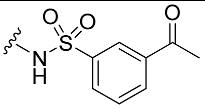	49 mg	39	99	509.1
**S5**	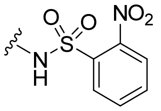	110 mg	86	99	512.1
**S6**	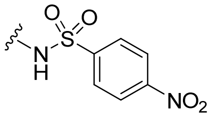	81 mg	63	99	512.1
**S7**	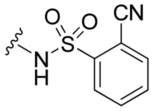	41 mg	33	99	492.1
**S8**	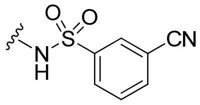	42 mg	35	99	492.1
**S9**	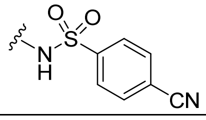	44 mg	36	99	492.1
**S10**	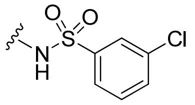	83 mg	66	99	501.1
**S11**	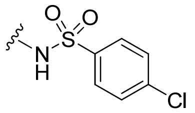	55 mg	44	99	501.1
**S12**	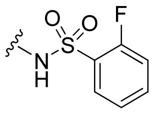	131 mg	72	99	485.1

Compound	R	Quantity	Yield (%)	Purity (%, HPLC-MS)	MS (ESI, $[M+H]^+$)
**S13**	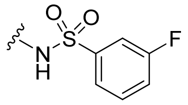	100 mg	82	99	485.1
**S14**	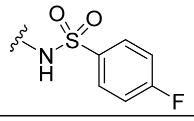	31 mg	25	99	485.1
**S15**	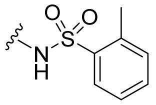	59 mg	49	99	481.1
**2**	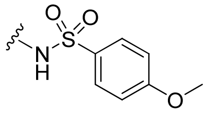	96 mg	77	98	497.1
**S16**	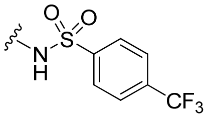	116 mg	86	99	535.1
**S17**	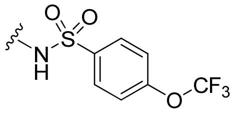	97 mg	71	99	551.1
**S18**	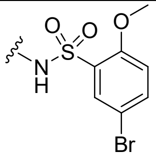	85 mg	59	99	575.1
**S19**	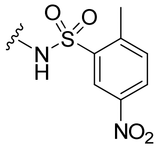	135 mg	68	99	526.1
**S20**	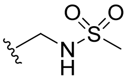	46 mg	46	98	418.4
**S21**	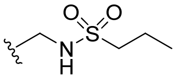	41 mg	38	98	447.2
**S22**	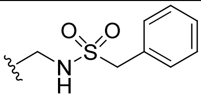	38 mg	32	94	495.2
**S23**	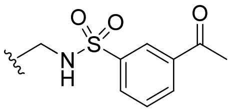	79 mg	62	99	523.1
**S24**	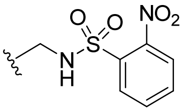	110 mg	58	98	526.1
**S25**	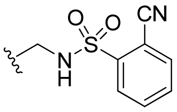	64 mg	53	99	506.1
**S26**	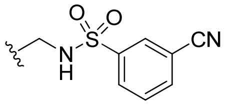	45 mg	37	98	506.1
**S27**	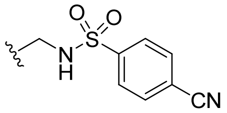	86 mg	70	98	506.1

Compound	R	Quantity	Yield (%)	Purity (%, HPLC-MS)	MS (ESI, $[M+H]^+$)
**S28**	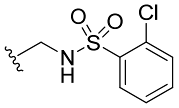	106 mg	57	99	515.1
**S29**	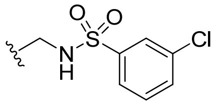	70 mg	57	97	515.1
**S30**	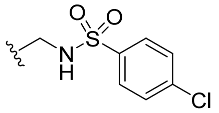	74 mg	59	95	515.1
**3**	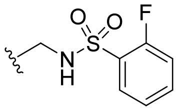	47 mg	39	99	499.2
**S31**	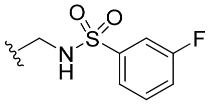	61 mg	51	97	499.2
**S32**	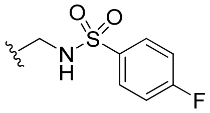	64 mg	53	97	499.2
**S33**	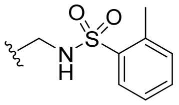	96 mg	53	95	495.2
**S34**	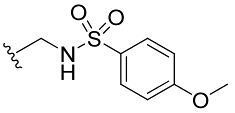	89 mg	72	98	511.2
**S35**	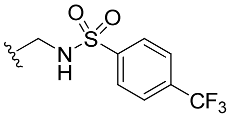	77 mg	58	98	549.1
**S36**	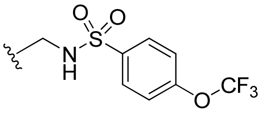	76 mg	55	97	565.1
**4**	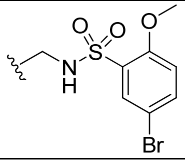	77 mg	54	99	589.1
**5**	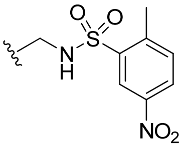	36 mg	27	99	540.1
**S37**	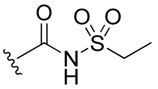	19 mg	15	99	447.1
**S38**	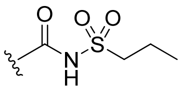	50 mg	39	99	461.1
**S39**[a]	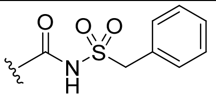	24 mg	13	99	509.1
**S40**	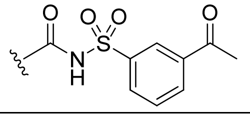	93 mg	62	98	537.1

Compound	R	Quantity	Yield (%)	Purity (%, HPLC-MS)	MS (ESI, $[M+H]^+$)
**S41**	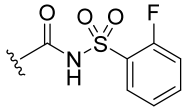	58 mg	40	99	513.1
**S42**	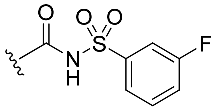	84 mg	58	99	513.1
**S43**	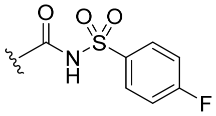	86 mg	59	99	513.1
**S44**	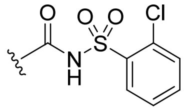	65 mg	44	99	529.1
**S45**	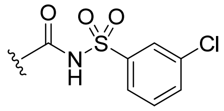	22 mg	15	99	529.1
**S46**	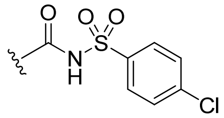	131 mg	88	99	529.1
**S47**	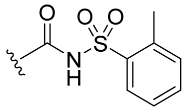	11 mg	8	99	509.1
**S48**	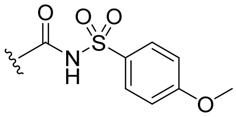	36 mg	24	99	525.1
**S49**	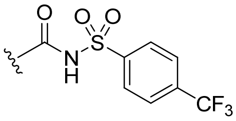	33 mg	21	99	563.1
**S50**	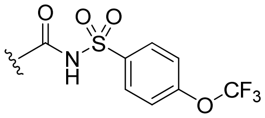	63 mg	39	97	579.1
**S51**	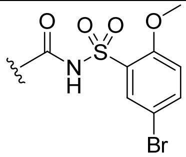	10 mg	6	99	603.1
**S52**	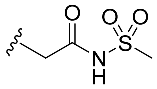	33 mg	27	99	447.1
**S53**	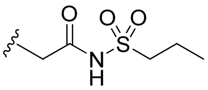	19 mg	15	99	475.1
**S54**	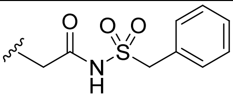	28 mg	20	99	523.2
**S55**	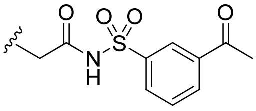	38 mg	25	99	551.1
**6**	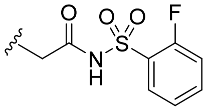	45 mg	31	99	527.1

Compound	R	Quantity	Yield (%)	Purity (%, HPLC-MS)	MS (ESI, [M+H]$^+$)
**S56**	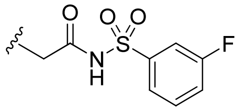	86 mg	61	99	527.1
**S57**	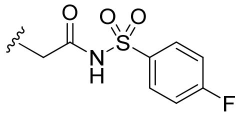	51 mg	36	99	527.1
**S58**	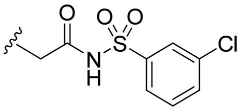	44 mg	30	99	543.1
**S59**	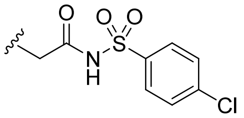	41 mg	28	99	543.1
**S60**	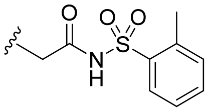	36 mg	26	95	523.1
**S61**	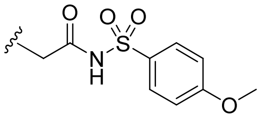	11 mg	8	98	539.1
**S62**	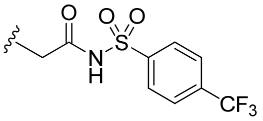	55 mg	36	99	577.1
**S63**	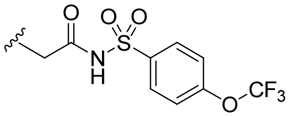	26 mg	16	99	593.1
**S64**	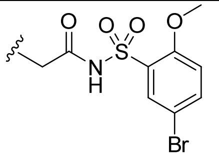	15 mg	9	98	617.1

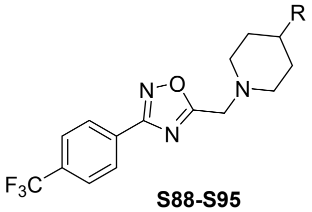

S88-S95

Compound	R	Quantity	Yield (%)	Purity (%, HPLC-MS)	MS (ESI, [M+H]$^+$)
**S88**	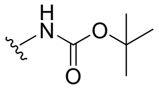	11.6 g	79	-	427.2
**S89**	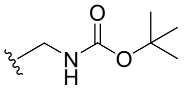	8 g	96	-	441.2
**S90**	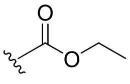	10.1 g	96	-	384.2
**S91**	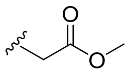	12.1 g	57	-	384.2
**S92**	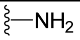	5 g	71	-	327.1

Compound	R	Quantity	Yield (%)	Purity (%, HPLC-MS)	MS (ESI, $[M+H]^+$)
**S93**	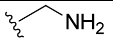	6.4 g	86	-	341.2
**S94**	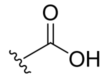	8 g	86	-	356.1
**S95**	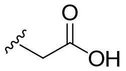	8 g	69	-	370.1

[a] The compound was purified by preparative HPLC [Phenomenex Lunc C18 10u (250 x 21.20 mm)] using 0.1 % TFA in water and methanol, and isolated as trifluoroacetate salt.

Table S2. Structure of commercial compounds.

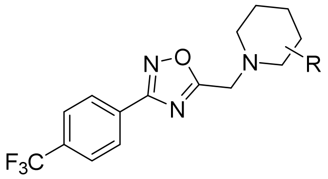

CAS number/PubChem CID	Position	R
**1147341-57-8**	4	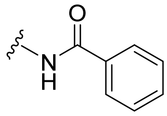
**1241585-33-0**	4	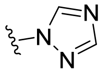
**1060474-28-3**	4	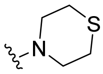
**1060379-06-7**	4	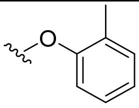
**1060547-43-4**	4	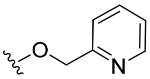
**1060922-96-4**	4	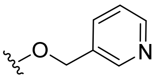
**1069949-01-4 (7)**	4	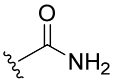
**1060413-50-4**	4	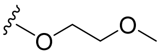
**1067025-98-2**	4	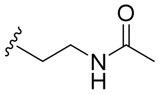
**878453-81-7**	3	—OH
**1070697-98-1**	3	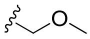
**1061079-84-2**	3	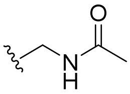
**1070757-29-7**	3	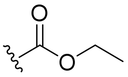

CAS number/PubChem CID	Position	R
**1067034-47-2**	3	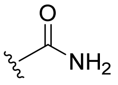
**1060820-64-5**	3	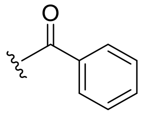
**1060509-13-8**	3	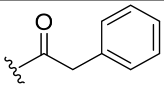
**1069673-30-8 (8)**	3	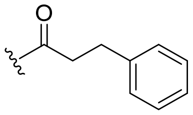
**45188906**	3	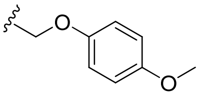
**1069551-91-2**	3	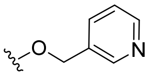
**878453-71-5 (9)**	3	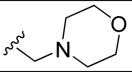
**1181512-54-8**	2	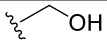
**45866530**	2	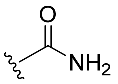
**1252086-33-1**	2	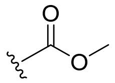
**1067006-98-7**	2	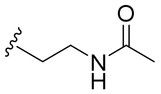
**45195410 (10)**	2	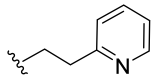
**45209972**	2	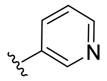
**45237175**	2	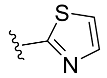

Table S3. Structure and synthetic data of compounds **S65-S87** and intermediates **S96-S118**.

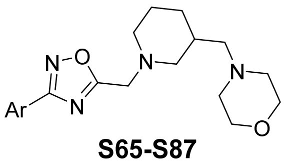

S65-S87

Compound	Ar	Quantity	Yield (%)	Purity (%, HPLC-MS)	MS (ESI, $[M+H]^+$)
**S65**	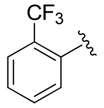	88 mg	86	99	411.2
**S66**	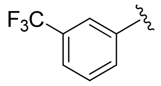	90 mg	88	98	411.2

Compound	Ar	Quantity	Yield (%)	Purity (%, HPLC-MS)	MS (ESI, $[M+H]^+$)
**S67**	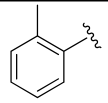	77 mg	86	99	357.2
**S68**	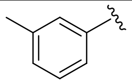	78 mg	88	99	357.2
**S69**	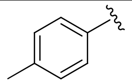	79 mg	89	99	357.2
**S70**	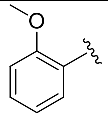	85 mg	91	99	373.2
**S71**	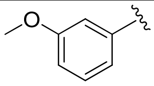	80 mg	86	99	373.2
**S72**	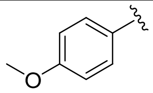	82 mg	88	99	373.2
**S73**	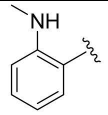	37 mg	40	97	372.2
**S74**	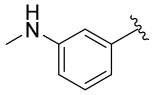	58 mg	62	99	372.2
**S75**	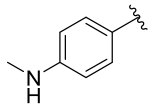	55 mg	59	96	372.2
**S76**	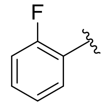	82 mg	91	99	361.2
**S77**	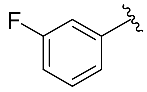	80 mg	89	99	361.2
**S78**	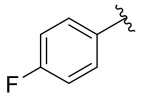	79 mg	88	99	361.2
**S79**	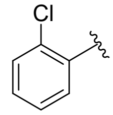	80 mg	85	95	377.2
**S80**	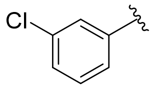	81 mg	86	95	377.2
**S81**	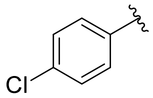	80 mg	85	99	377.2
**S82**	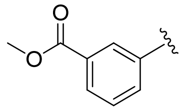	81 mg	81	99	401.2
**S83**	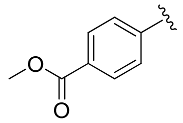	83 mg	83	99	401.2

Compound	Ar	Quantity	Yield (%)	Purity (%, HPLC-MS)	MS (ESI, $[M+H]^+$)
**S84**	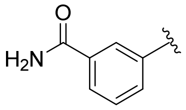	81 mg	84	99	386.2
**S85**	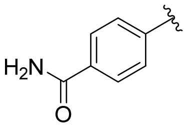	79 mg	82	95	386.2
**S86**	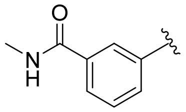	83 mg	83	99	400.2
**S87**	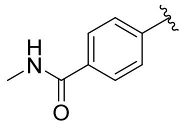	86 mg	86	98	400.2

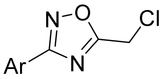

S96-S118

Compound	Ar	Quantity	Yield (%)	Purity (%, HPLC-MS)	MS (ESI, $[M+H]^+$)
**S96**	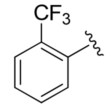	193 mg	49	-	263.1
**S97**	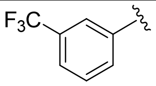	177 mg	45	-	263.1
**S98**	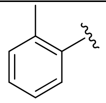	156 mg	50	-	209.1
**S99**	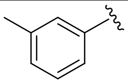	144 mg	46	-	209.1
**S100**	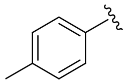	166 mg	53	-	209.1
**S101**	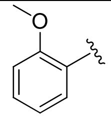	185 mg	55	-	225.1
**S102**	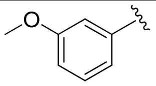	175 mg	52	-	225.1
**S103**	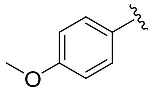	155 mg	46	-	225.1
**S104**	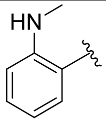	91 mg	27	-	224.0
**S105**	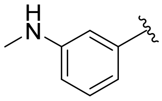	111 mg	33	-	224.0
**S106**	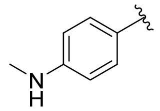	118 mg	35	-	224.0

Compound	Ar	Quantity	Yield (%)	Purity (%, HPLC-MS)	MS (ESI, $[M+H]^+$)
**S107**	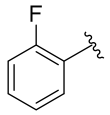	137 mg	43	-	213.0
**S108**	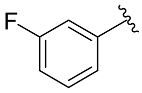	150 mg	47	-	213.0
**S109**	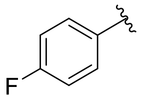	131 mg	41	-	213.0
**S110**	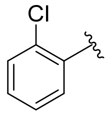	168 mg	49	-	229.0
**S111**	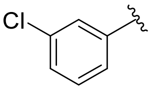	155 mg	45	-	229.0
**S112**	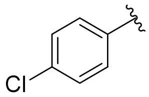	131 mg	38	-	229.0
**S113**	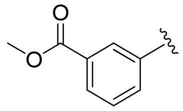	159 mg	42	-	253.1
**S114**	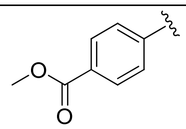	170 mg	45	-	253.1
**S115**	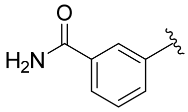	157 mg	44	-	238.0
**S116**	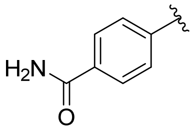	167 mg	47	-	238.0
**S117**	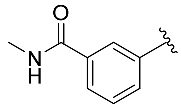	147 mg	39	-	252.0
**S118**	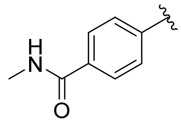	162 mg	43	-	252.0

2. NMR spectra and HPLC trace analysis of compounds 7-10

^{1}H NMR spectrum for **7** (DMSO-*d6*, 300 MHz)

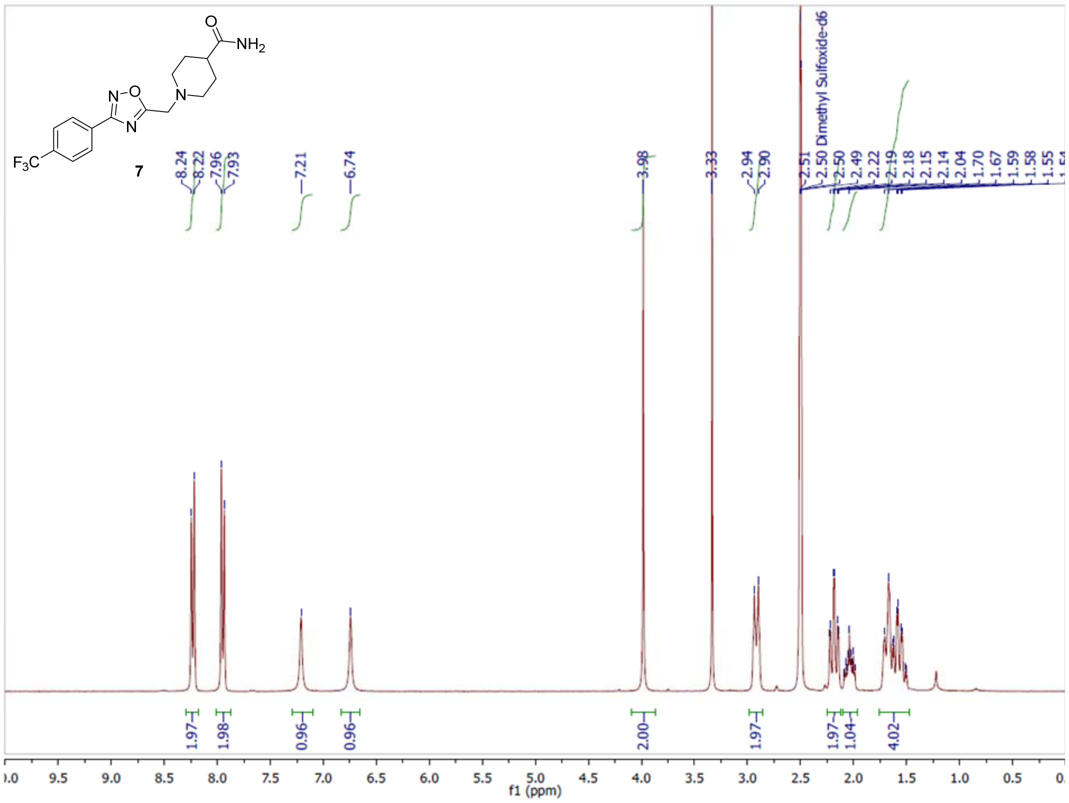

^{13}C NMR spectrum for **7** (DMSO-*d6*, 75 MHz)

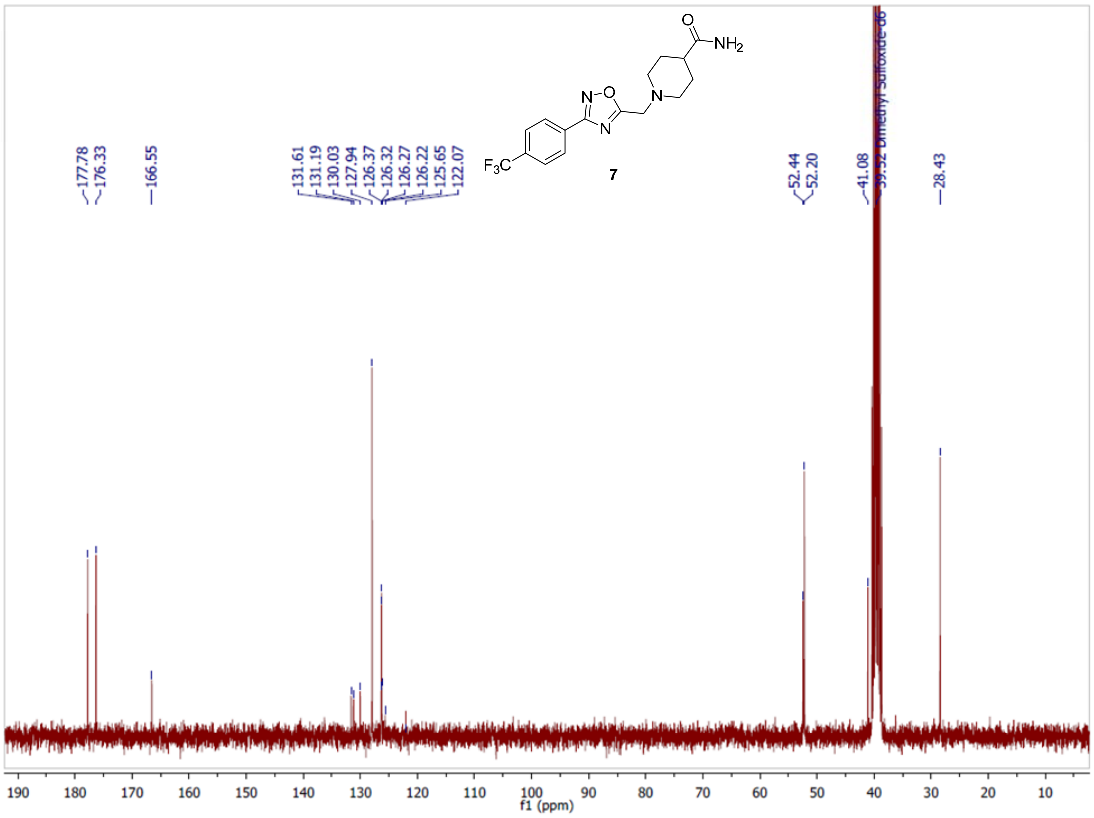

^{1}H NMR spectrum for **8** ($CDCl_3$, 300 MHz)

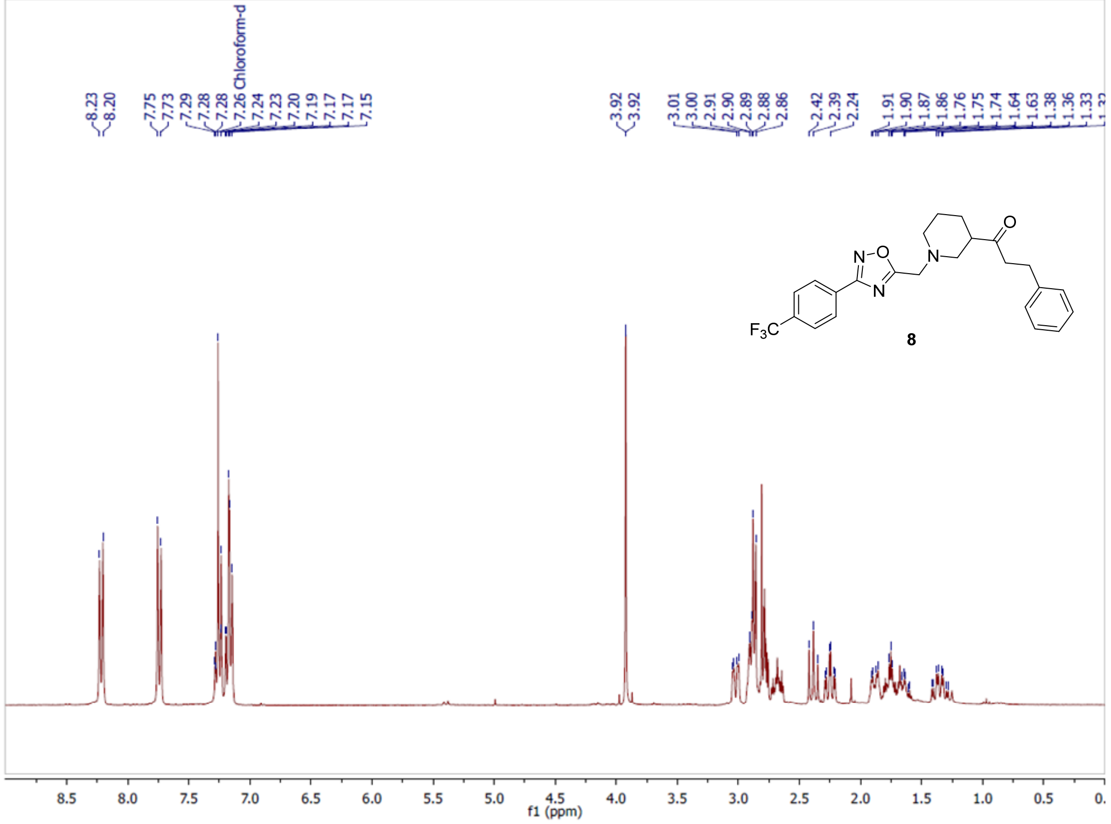

^{13}C NMR spectrum for **8** ($CDCl_3$, 75 MHz)

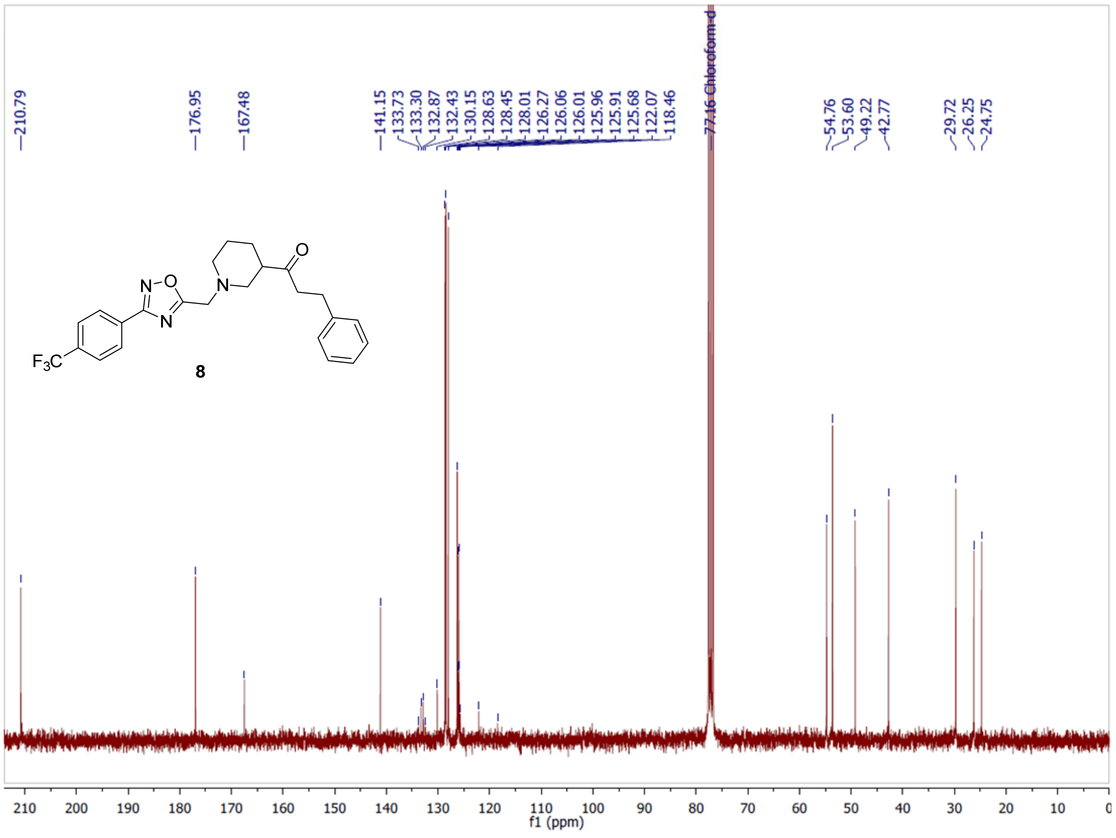

^{1}H NMR spectrum for **9** ($CDCl_3$, 300 MHz)

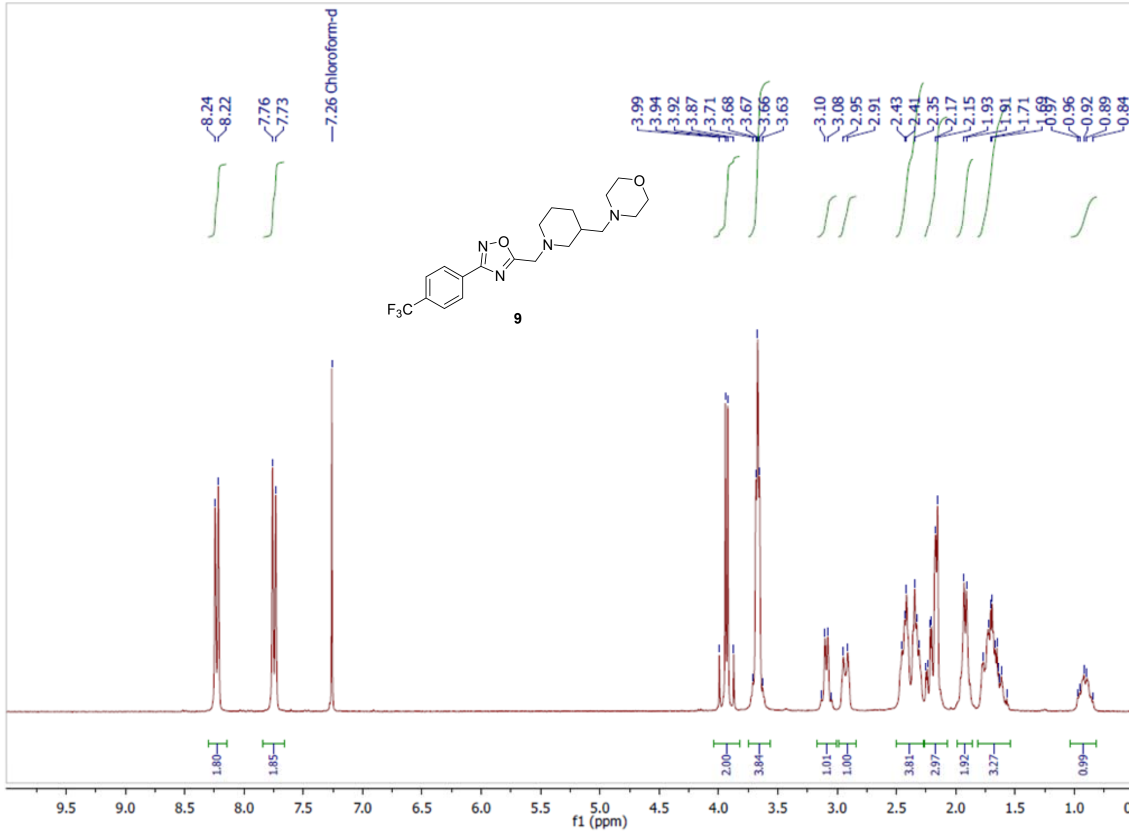

^{13}C NMR spectrum for **9** ($CDCl_3$, 75 MHz)

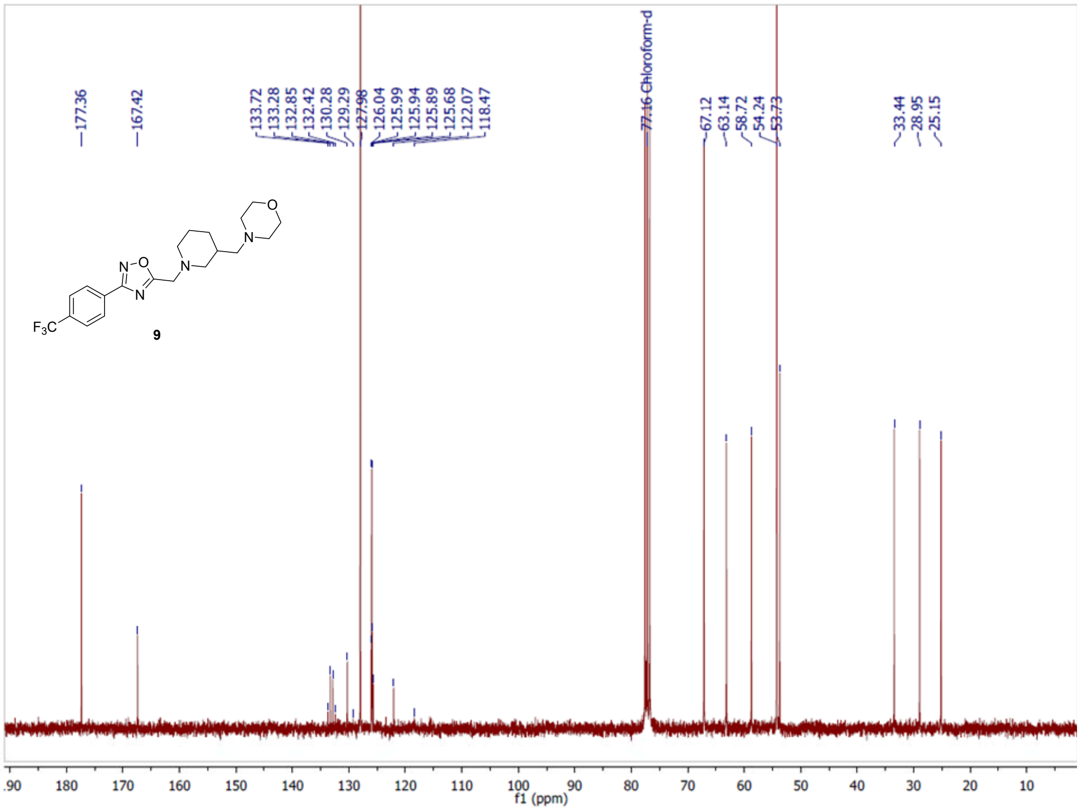

^{1}H NMR spectrum for **10** ($CDCl_3$, 300 MHz)

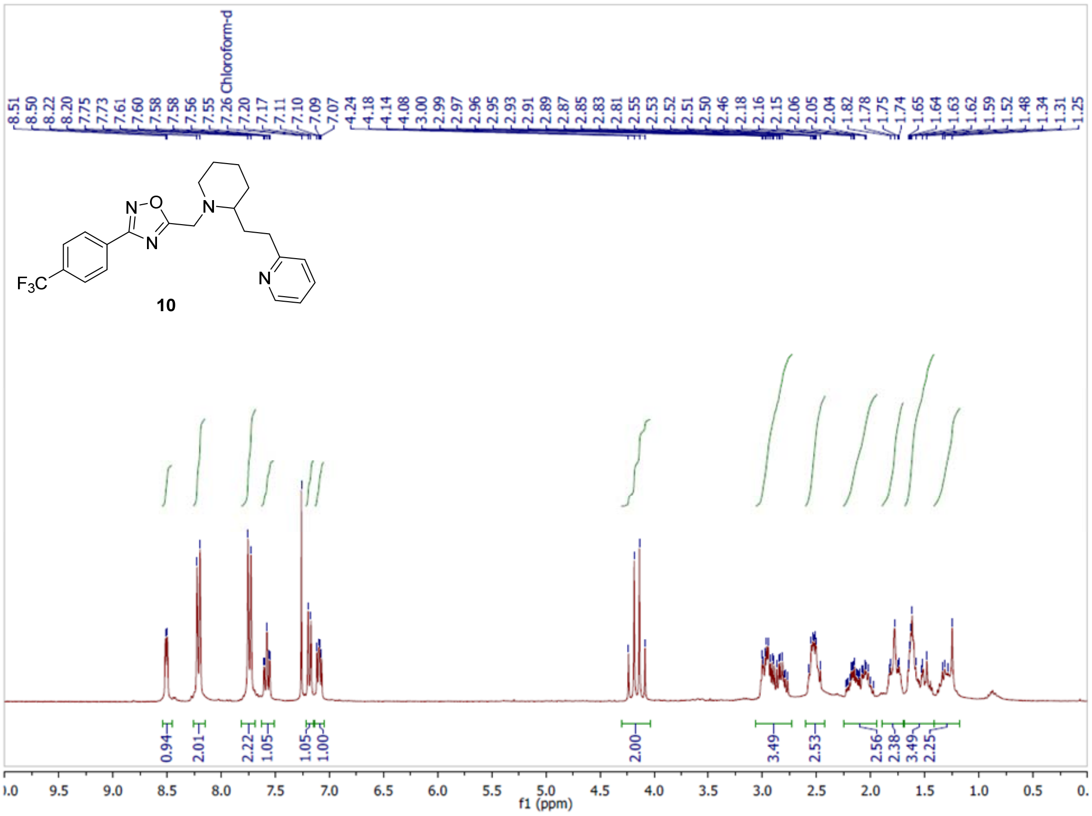

^{13}C NMR spectrum for **10** ($CDCl_3$, 75 MHz)

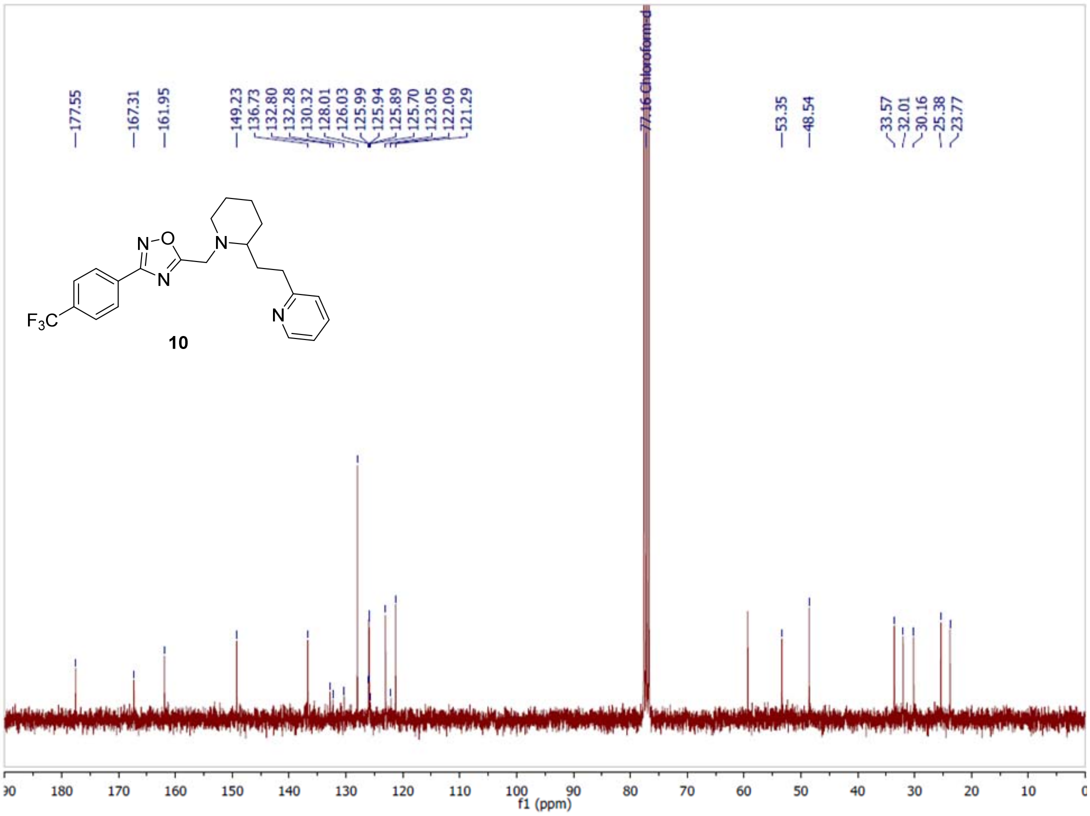

HPLC traces:

Compound **7**:

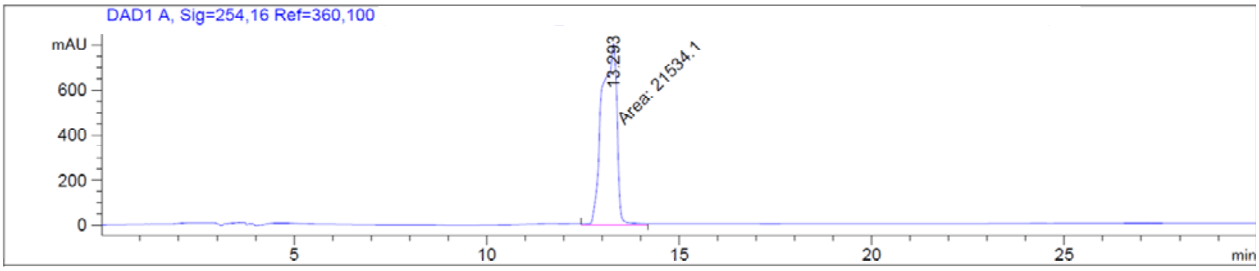

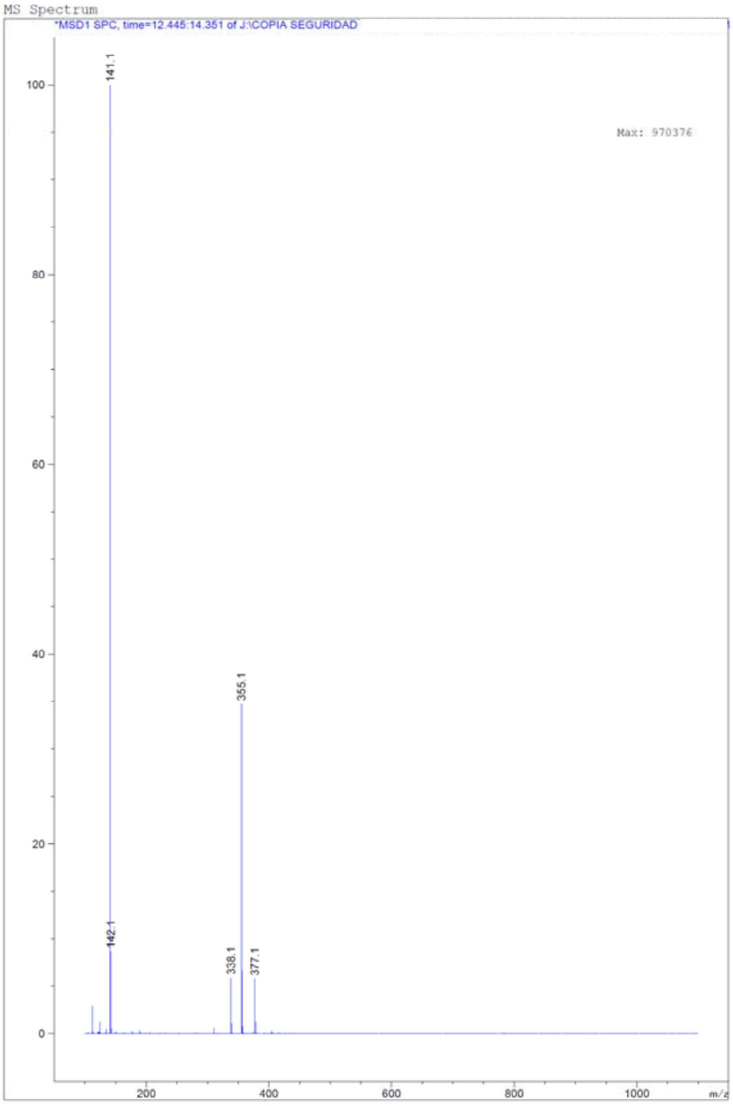

Compound **8**:

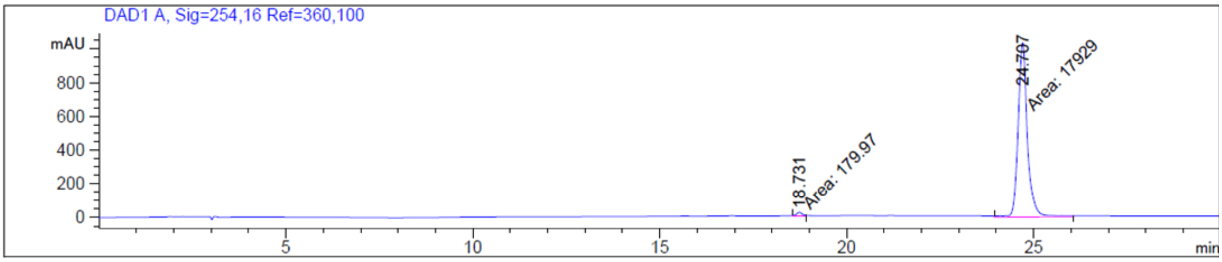

Peak #	RetTime [min]	Type	Width [min]	Area [mAU*s]	Height [mAU]	Area %
1	18.731	MM	0.1577	179.97037	19.02028	0.9938
2	24.707	MM	0.2890	1.79290e4	1033.99719	99.0062
Totals :				1.81089e4	1053.01747	

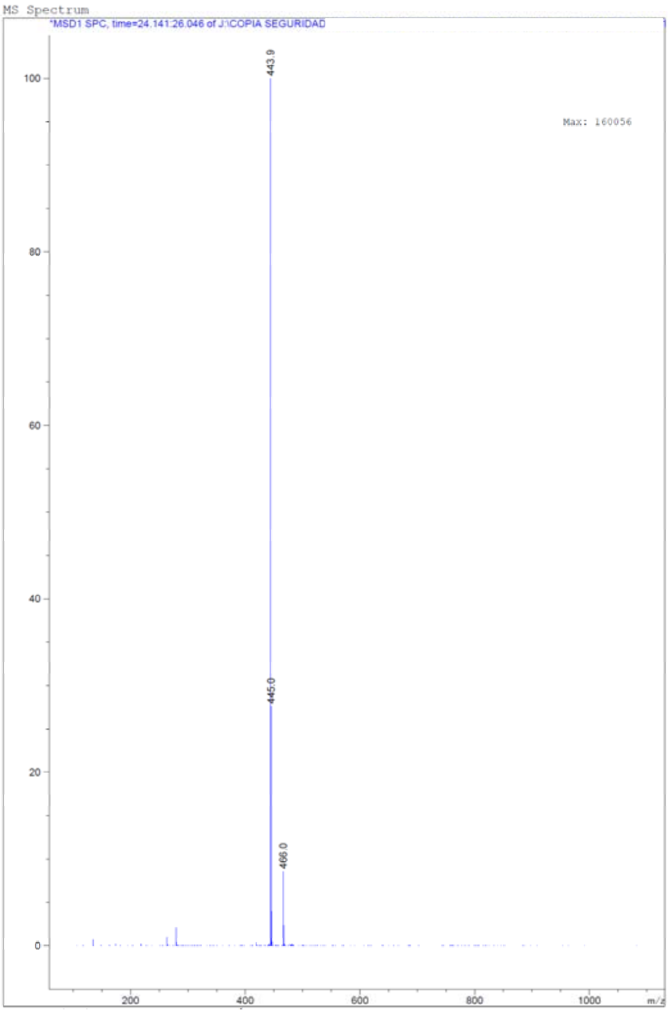

rac-**9**:

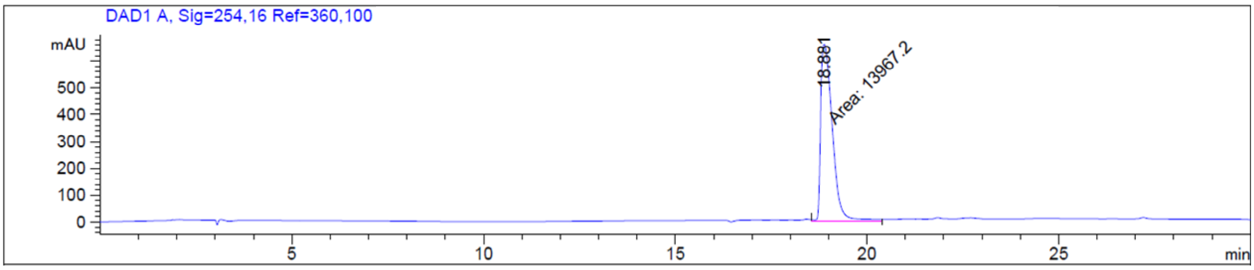
DAD1 A, Sig=254,16 Ref=360,100
mAU
500
400
300
200
100
0
18.881
Area: 13967.2
5
10
15
20
25
min

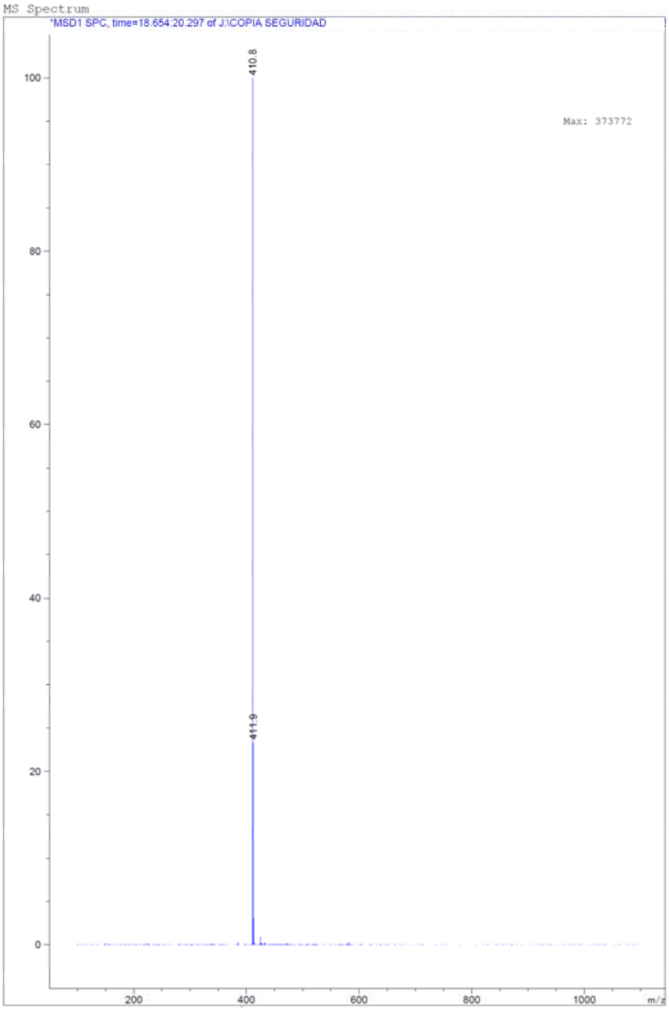
MS Spectrum
*MSD1 SPC, time=18.654:20.297 of J:\COPIA SEGURIDAD
410.8
411.9
Max: 373772
100
80
60
40
20
0
200
400
600
800
1000
m/z

(*S*)-(+)-**9**:

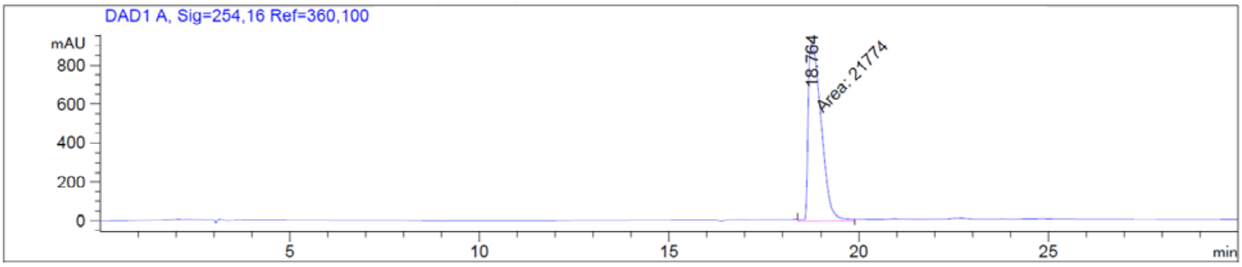
DAD1 A, Sig=254,16 Ref=360,100
mAU
800
600
400
200
0
5
10
15
20
25
min
18.764
Area: 21774

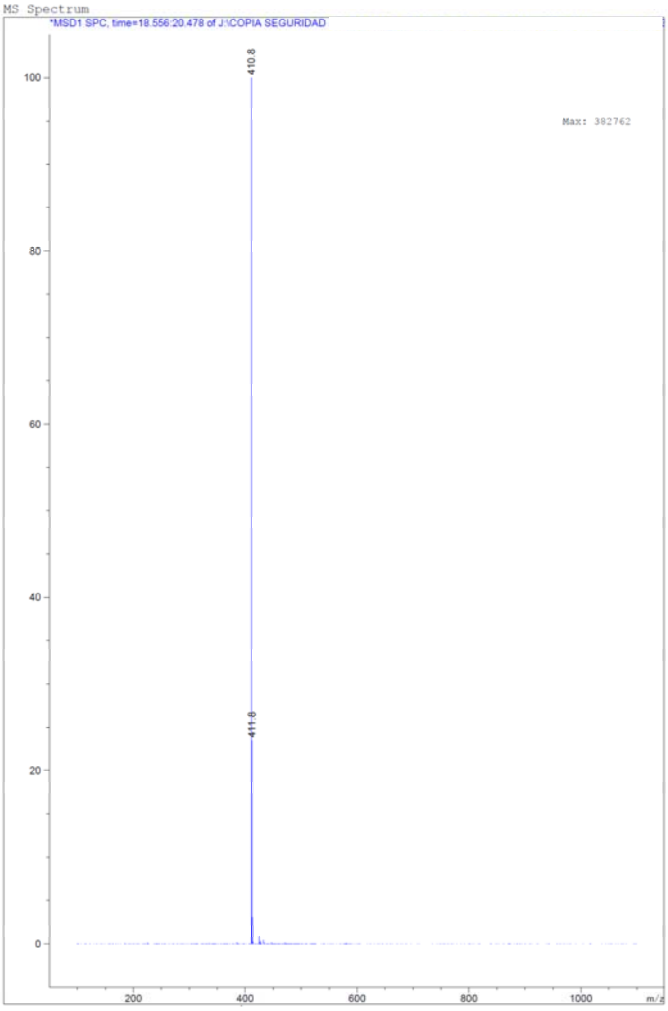
MS Spectrum
*MSD1 SPC, time=18.556:20.478 of J:\COPIA SEGURIDAD
410.8
411.8
Max: 382762
100
80
60
40
20
0
200
400
600
800
1000
m/z

(*R*)-(−)-**9**:

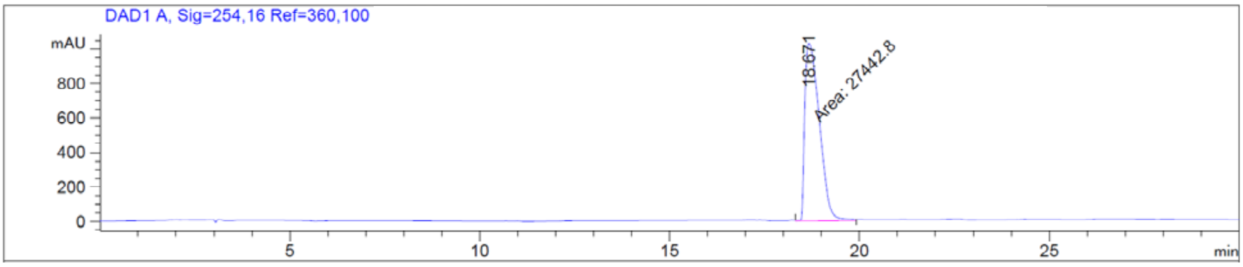
DAD1 A, Sig=254,16 Ref=360,100
mAU
800
600
400
200
0
18.671
Area: 27442.8
5
10
15
20
25
min

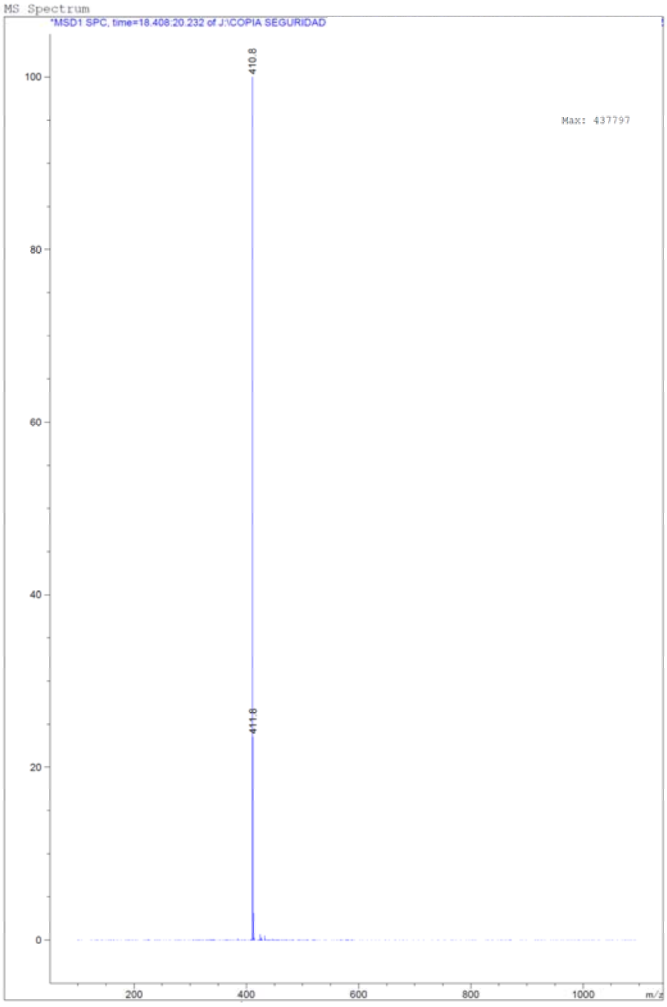
MS Spectrum
*MSD1 SPC, time=18.408:20.232 of J:\COPIA SEGURIDAD
410.8
411.8
Max: 437797
100
80
60
40
20
0
200
400
600
800
1000
m/z

Compound **10**:

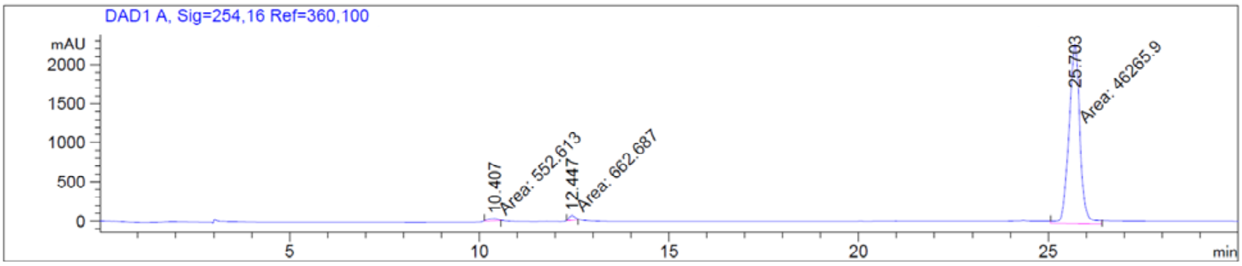

Peak #	RetTime [min]	Type	Width [min]	Area [mAU*s]	Height [mAU]	Area %
1	10.407	MM	0.3186	552.61261	28.90401	1.1639
2	12.447	MM	0.1846	662.68719	59.82944	1.3957
3	25.703	MM	0.3350	4.62659e4	2301.72827	97.4405
Totals :				4.74812e4	2390.46173	

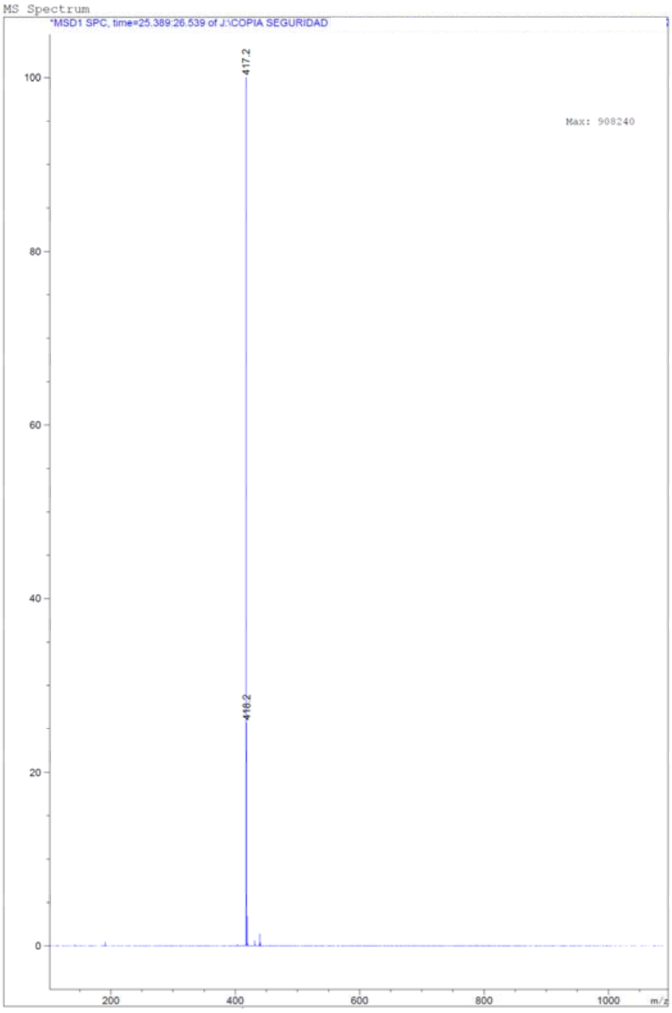

Chiral HPLC traces:

rac-**9**:

```
Sample Info      : HEXANO/ISOPROPANOL 90/10 1.1 ML/MIN 44 Bar
                   COLUMNA IA
```

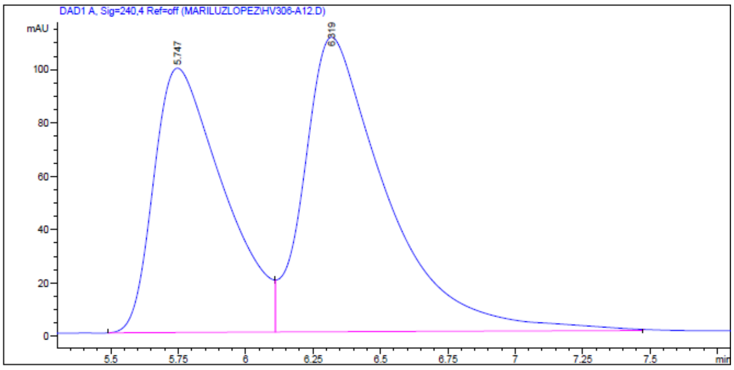


```
=====================================================================
                         Area Percent Report
=====================================================================

Sorted By             :      Signal
Multiplier            :      1.0000
Dilution              :      1.0000
Use Multiplier & Dilution Factor with ISTDs


Signal 1: DAD1 A, Sig=240,4 Ref=off

Peak RetTime Type  Width     Area      Height     Area
  #   [min]        [min]   [mAU*s]     [mAU]        %
----|-------|----|-------|----------|----------|--------|
   1   5.747 BV     0.2668 1791.63623   99.16078  43.1554
   2   6.319 VB     0.3012 2359.95142  110.47558  56.8446

Totals :                   4151.58765  209.63636
```

(*S*)-(+)-**9**:

```
Sample Info      : HEXANO/ISOPROPANOL 90/10 1.1 ML/MIN 44 Bar
                   COLUMNA IA
```

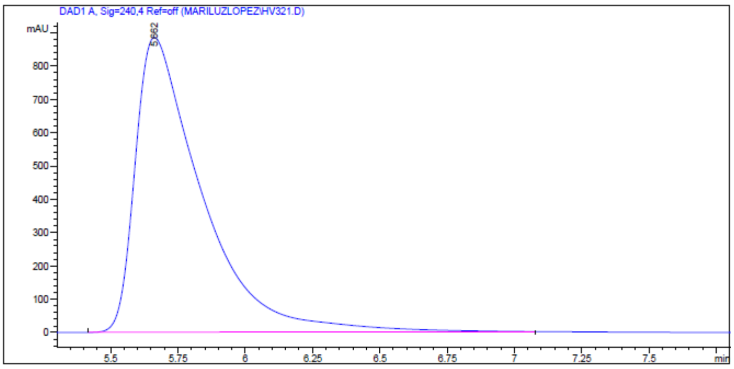


```
=====================================================================
                         Area Percent Report
=====================================================================

Sorted By             :      Signal
Multiplier            :      1.0000
Dilution              :      1.0000
Use Multiplier & Dilution Factor with ISTDs


Signal 1: DAD1 A, Sig=240,4 Ref=off

Peak RetTime Type  Width     Area      Height     Area
  #   [min]        [min]   [mAU*s]     [mAU]        %
----|-------|----|-------|----------|----------|--------|
   1   5.662 BB     0.2455 1.53033e4  886.03357 100.0000

Totals :                   1.53033e4  886.03357
```

(*R*)-(−)-**9**:

```
Sample Info      : HEXANO/ISOPROPANOL 90/10 1.1 ML/MIN 45 Bar
                   COLUMNA IA
```

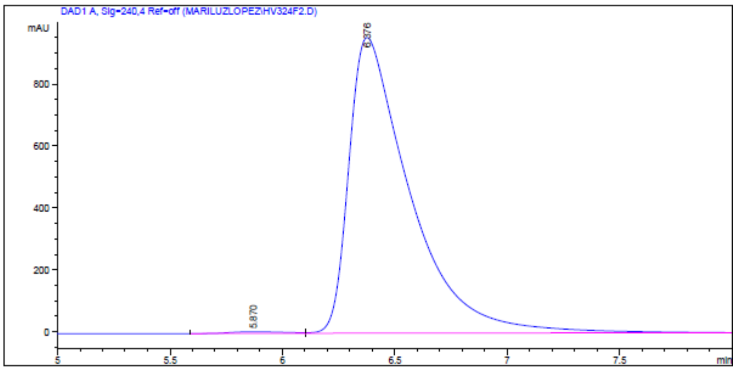


```
=====================================================================
                         Area Percent Report
=====================================================================

Sorted By             :      Signal
Multiplier            :      1.0000
Dilution              :      1.0000
Use Multiplier & Dilution Factor with ISTDs


Signal 1: DAD1 A, Sig=240,4 Ref=off

Peak RetTime Type  Width     Area      Height     Area
  #   [min]        [min]   [mAU*s]     [mAU]        %
----|-------|----|-------|----------|----------|--------|
   1   5.870 BV    0.2566   96.44057    5.78297   0.5286
   2   6.376 VB    0.2738 1.81486e4  954.64124  99.4714

Totals :                  1.82450e4  960.42420

=====================================================================
                          *** End of Report ***
```

3. Binding to off-target receptors

Selectivity of compound **9** was analyzed in a panel of binding assays toward 54 receptors in Eurofins Cerep (France). Significant binding affinity was defined as a displacement of target selective radioligand greater than 50% using a concentration of 10 µM of **9**.

Table S4. Selectivity panel of compound **9**.

Assay<br>Cerep Compound I.D.	Client Compound I.D.	Test Concentration (M)	% Inhibition of Control Specific Binding
**A_1 *(h)* (antagonist radioligand)**			
20815-1	C6	1.0E-05	5
**A_{2A} *(h)* (agonist radioligand)**			
20815-1	C6	1.0E-05	-3
**A_3 *(h)* (agonist radioligand)**			
20815-1	C6	1.0E-05	-20
**α_1 (non-selective) (antagonist radioligand)**			
20815-1	C6	1.0E-05	1
**α_2 (non-selective) (antagonist radioligand)**			
20815-1	C6	1.0E-05	9
**β_1 *(h)* (agonist radioligand)**			
20815-1	C6	1.0E-05	3
**β_2 *(h)* (agonist radioligand)**			
20815-1	C6	1.0E-05	-11
**AT_1 *(h)* (antagonist radioligand)**			
20815-1	C6	1.0E-05	-6
**BZD (central) (agonist radioligand)**			
20815-1	C6	1.0E-05	-17
**B_2 *(h)* (agonist radioligand)**			
20815-1	C6	1.0E-05	8
**CB_1 *(h)* (agonist radioligand)**			
20815-1	C6	1.0E-05	15
**CCK_1 (CCK_A) *(h)* (agonist radioligand)**			
20815-1	C6	1.0E-05	2
**D_1 *(h)* (antagonist radioligand)**			
20815-1	C6	1.0E-05	4
**D_{2S} *(h)* (antagonist radioligand)**			
20815-1	C6	1.0E-05	8
**ET_A *(h)* (agonist radioligand)**			
20815-1	C6	1.0E-05	-13
**GABA (non-selective) (agonist radioligand)**			
20815-1	C6	1.0E-05	9
**GAL_2 *(h)* (agonist radioligand)**			
20815-1	C6	1.0E-05	0
**CXCR2 (IL-8B) *(h)* (agonist radioligand)**			
20815-1	C6	1.0E-05	-6
**CCR1 *(h)* (agonist radioligand)**			
20815-1	C6	1.0E-05	4
**H_1 *(h)* (antagonist radioligand)**			
20815-1	C6	1.0E-05	-4
**H_2 *(h)* (antagonist radioligand)**			
20815-1	C6	1.0E-05	-13
**MC_4 *(h)* (agonist radioligand)**			
20815-1	C6	1.0E-05	-8
**MT_1 (ML_{1A}) *(h)* (agonist radioligand)**			
20815-1	C6	1.0E-05	-3
**M_1 *(h)* (antagonist radioligand)**			
20815-1	C6	1.0E-05	30
**M_2 *(h)* (antagonist radioligand)**			
20815-1	C6	1.0E-05	-7
**M_3 *(h)* (antagonist radioligand)**			
20815-1	C6	1.0E-05	45
**NK_2 *(h)* (agonist radioligand)**			
20815-1	C6	1.0E-05	19
**NK_3 *(h)* (antagonist radioligand)**			
20815-1	C6	1.0E-05	-8

Assay<br>Cerep Compound I.D.	Client Compound I.D.	Test Concentration (M)	% Inhibition of Control Specific Binding
**Y_1 *(h)* (agonist radioligand)**			
20815-1	C6	1.0E-05	-9
**Y_2 *(h)* (agonist radioligand)**			
20815-1	C6	1.0E-05	-20
**NTS_1 (NT_1) *(h)* (agonist radioligand)**			
20815-1	C6	1.0E-05	3
**δ_2 (DOP) *(h)* (agonist radioligand)**			
20815-1	C6	1.0E-05	2
**κ (KOP) (agonist radioligand)**			
20815-1	C6	1.0E-05	7
**μ (MOP) *(h)* (agonist radioligand)**			
20815-1	C6	1.0E-05	5
**NOP (ORL1) *(h)* (agonist radioligand)**			
20815-1	C6	1.0E-05	1
**EP_4 *(h)* (agonist radioligand)**			
20815-1	C6	1.0E-05	4
**5-HT_{1A} *(h)* (agonist radioligand)**			
20815-1	C6	1.0E-05	0
**5-HT_{1B} (antagonist radioligand)**			
20815-1	C6	1.0E-05	-7
**5-HT_{2A} *(h)* (antagonist radioligand)**			
20815-1	C6	1.0E-05	26
**5-HT_{2B} *(h)* (agonist radioligand)**			
20815-1	C6	1.0E-05	35
**5-HT_3 *(h)* (antagonist radioligand)**			
20815-1	C6	1.0E-05	-1
**5-HT_{5a} *(h)* (agonist radioligand)**			
20815-1	C6	1.0E-05	-3
**5-HT_6 *(h)* (agonist radioligand)**			
20815-1	C6	1.0E-05	-3
**5-HT_7 *(h)* (agonist radioligand)**			
20815-1	C6	1.0E-05	-7
**sst (non-selective) (agonist radioligand)**			
20815-1	C6	1.0E-05	-9
**$VPAC_1$ (VIP_1) *(h)* (agonist radioligand)**			
20815-1	C6	1.0E-05	-3
**V_{1a} *(h)* (agonist radioligand)**			
20815-1	C6	1.0E-05	8
**Ca^{2+} channel (L, verapamil site) (phenylalkylamine) (antagonist radioligand)**			
20815-1	C6	1.0E-05	0
**K_V channel (antagonist radioligand)**			
20815-1	C6	1.0E-05	-2
**SK_{Ca} channel (antagonist radioligand)**			
20815-1	C6	1.0E-05	-12
**Cl^- channel (GABA-gated) (antagonist radioligand)**			
20815-1	C6	1.0E-05	12
**norepinephrine transporter *(h)* (antagonist radioligand)**			
20815-1	C6	1.0E-05	5
**dopamine transporter *(h)* (antagonist radioligand)**			
20815-1	C6	1.0E-05	4
**5-HT transporter *(h)* (antagonist radioligand)**			
20815-1	C6	1.0E-05	6

4. Pharmacokinetics of compound 9

In-vitro membrane permeability, microsomal stability (rat, dog, and human liver microsomes), inhibition of P450 cytochrome isoforms (1A2, 2C9, 2C19, 2D6 and 3A), effect on human ether-à-go-go related gene (hERG) channel as well as oral and intravenous pharmacokinetics of **9** were performed by Eurofins Advinus (India), according to the following procedures.

In-vitro membrane permeability. The assessment of the membrane permeability of compound **9** was performed using the PAMPA-BBB method, which employs a polar brain lipid (PBL) porcine membrane. The assay was conducted in PBS pH 7.4 buffer and the compound was tested at 50 and 100 µM. The polyvinylidene fluoride filter membrane of the donor plate was first coated with 4 µL of PBL in dodecane (20 mg/mL) (from Avanti Polar Lipids), and the acceptor plate well was filled with 300 µL of PBS. Compound **9** was dissolved in DMSO and diluted with PBS to reach the corresponding final concentration (50 or 100 µM) in the donor plate well (DMSO concentration below 0.5% v/v). An aliquot of 300 µL of compound solution was added to the donor plate well and this plate was carefully placed into the acceptor plate. The assembly was incubated for 5 h at 25 °C to allow the diffusion of test compound from the donor to the acceptor well through the lipid membrane. After incubation, the donor plate was carefully removed, and the concentration of compound **9** in both donor and acceptor wells was determined by UV spectroscopy (wavelength set at 240 nm). In addition, a compound solution of a theoretical concentration that simulated the equilibrium state established if the membrane were ideally permeable was assayed. Concentration of compound **9** in both donor and acceptor wells and equilibrium concentration were determined by using a standard curve. The effective permeability value (Pe) was calculated using the following equation: Pe (cm s^{-1}) = $C \cdot \ln(1 - C_A / C_{eq})$, where $C = (V_A \cdot V_D)/[(V_A + V_D) \cdot A \cdot t]$, where A = filter well area (0.3 cm^2), V_A = acceptor well volume (0.3 mL), V_D = donor well volume (0.3 mL), t = incubation time (18000 s), C_A = compound concentration (µM) in acceptor well, C_D = compound concentration (µM) in donor well, and C_{eq} = equilibrium concentration. Each concentration tested for compound **9** was measured in triplicate. Verapamil (high permeability, Pe = $11.4 \cdot 10^{-6}$ cm s^{-1}) and atenolol (low permeability, Pe = $0.3 \cdot 10^{-6}$ cm s^{-1}) were used as control compounds and met the acceptance criteria.

Stability assays in rat, dog and human liver microsomes. Compound **9** or verapamil (used as control) were incubated at 37 °C in PBS (1 µM) together with a solution of NADPH in PBS (2 mM) and a solution of $MgCl_2$ in PBS (5 mM). Metabolic reactions were initiated by the addition of a suspension of rat, dog or human liver microsomes at a final protein concentration of 1 mg/mL. The

solutions were shaken in a vortex and kept in a water bath at 37 °C. Aliquots of 100 μL were quenched at time zero and at seven points ranging to 1 h by pouring into 100 μL of ice-cold acetonitrile. Quenched samples were centrifuged at 10000*g* for 5 min, and the supernatants were filtered through a polytetrafluoroethylene membrane syringe filter (pore size of 0.2 μm). The relative loss of parent compound over the course of the incubation was monitored by HPLC−MS. Concentrations were quantified by measuring the area under the peak ($M + H^+$) and converted to the percentage of compound remaining, using the time zero peak area value as 100%. The natural logarithm of the percentage remaining versus time data for each compound was fit to linear regression, and the slope was used to calculate the degradation half-life time. The latter was then used to calculate the intrinsic plasma clearance (CL_{int}).

Inhibition of P450 cytochrome isoforms. In-vitro incubation to determine the potential of compound **9** to reversibly inhibit human P450 cytochrome (CYP) enzymes was performed using the recombinantly expressed CYP isoforms 1A2, 2C9, 2C19, 2D6, and 3A. Specific substrates were used for each isoenzyme, which were known to be selectively metabolized to fluorescent metabolites. Test compound was incubated with individual isoenzymes over a concentration range up to 10 μM. After incubation, fluorescence level was measured on a plate reader. Fluorescence values in the absence and presence of test compound was used to calculate the IC_{50} against each CYP isoform.

Effect on hERG channel. The inhibitory effect of compound **9** on the potassium-selective IKr (tail) current was investigated using Chinese Hamster Ovary (CHO) cells stably transfected with hERG, employing the whole cell patch clamp technique. Compound **9** was tested at 1, 10 and 30 μM, using 0.1 % v/v DMSO in extracellular (EC) solution as vehicle control, and propafenone hydrochloride at 10 μM was used as positive control to confirm the sensitivity of test system to hERG inhibition. Patch clamp experiments were carried out using FlyscreenP® robot. CHO cells were stably transfected with the hERG clone (hERG-TRex-CHO) and maintained at 35 °C in a 5 % CO_2 / 95 % air incubator. The cells were subcultured 24-48 h before hERG induction, which was carried out for 18-36 h with doxycycline (3 μg/mL) before start of patch clamp experiments. After

the induction, cells were dislodged and suspended in EC solution with choline chloride (145 mM choline chloride, 5 mM KCl, 1 mM $MgCl_2(H_2O)_6$, 10 mM HEPES, 10 mM glucose, pH 7.4) to give cell density of ~ 0.5 million per mL, and transferred to the cell mixer on Flyscreen® 8500 robot which keeps cell suspension in a ready-for-use condition. Intracellular solution consisted of 115 mM $KMeSO_3$, 5 mM $MgCl_2(H_2O)_6$, 10 mM HEPES-KOH, 5 mM EGTA, 5 mM K_2ATP at pH 7.2. Ionic currents from hERG transfected cells were measured in the whole cell configuration. Once the whole cell configuration was achieved, test compound **9** was added to the cells for recording the effect on the hERG current. An EC NaCl solution (120 mM NaCl, 5 mM KCl, 1.8 mM $CaCl_2$, 10 mM HEPES, 10 mM glucose, pH 7.4) was added for control readings, which were measured for ~ 360 s followed by compound addition. The effect of compound **9** on tail current was monitored continuously for ~ 600 seconds. For analysis purposes, the current of interest (IKr) was considered. Results were expressed as absolute values and percent change from control. Percent inhibition was calculated by comparing the IKr current before and after addition of test compound once a steady-state current had been attained.

In-vivo pharmacokinetic study of compound 9. The study was conducted in two groups each containing four male Sprague Dawley rats with a parallel design following iv and oral administration. Vehicle (0.2% DMSO in saline) or solution formulation prepared for compound **9** was administered to the rats (2 mg/kg, iv and 10 mg/kg, po), and blood samples were collected at pre-dose (0 h), 0.083 (iv only) 0.25, 0.5, 1, 2, 4, 6, 8 and 24 h post-dose. K_2EDTA was used as an anti-coagulant. Plasma samples were collected by centrifugation at 5000*g* for 10 min at 4 ºC. Fit-for purpose LC-MS/MS method was used for the quantification of compound **9** in rat plasma samples. The lower limit of quantification of the bioanalytical method used for compound **9** in rat plasma samples was 5.03 ng/mL. Plasma samples were stored at -80 ºC until their analysis. Pharmacokinetic parameters were calculated using the non-compartmental analysis tool of WinNonlin validated software (version 5.2) and were determined for each group. The area under the plasma concentration-time curve ($AUC_{0\text{-}t}$ and $AUC_{0\text{-}inf}$), elimination half-life ($T_{1/2}$), clearance (CL) and volume of distribution (V_{ss}) were calculated from intravenous group. The peak plasma concentration

(C_{max}), time to achieve peak plasma concentration (T_{max}), $AUC_{0\text{-}t}$ and $AUC_{0\text{-}inf}$, and oral bioavailability (F) were calculated from the oral group.

Table S5. Brain penetration of compound **9** in a PAMPA-BBB assay.

Concentration (μM)	Pe (cm/s)
50	2.0 x 10^{-6}
100	1.8 x 10^{-6}

Table S6. Microsomal stability of compound **9**.

	9			Verapamil		
	Human	Dog	Rat	Human	Dog	Rat
**% Metabolism after 30 min**	39	46	42	74	87	78
**Half-life time (min)**	>30	>30	>30	13	8	12
**Cl_{int} (mL/min/g liver)**	1.8	2.3	2.1	5	9	6

Table S7. P450 Cytochrome inhibition of compound **9**.

**Isoenzyme**	**IC_{50} (μM)**	
	**9**	**Positive Control**
CYP1A2	26	0.008
CYP2C9	80	0.45
CYP2C19	48	0.59
CYP2D6	19	0.08
CYP3A	>100	0.011

Table S8. hERG Channel inhibition of compound **9**.

	**Concentration (μM)**	**Inhibition (%)**		**Mean**
**9**[a]	1	27.0	32.3	29.7
	10	52.1	72.5	62.3
	30	78.0	83.0	80.5
**Propafenone**	10	71.0	80.5	75.7
**Vehicle**	0	0.0	10.0	5.0

[a] IC_{50} = 4.129 μM

5. Figures

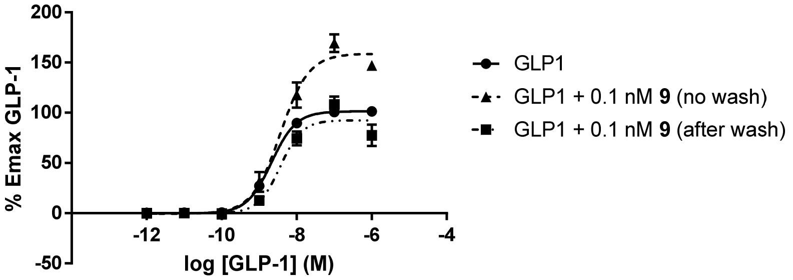

Figure S1. The potentiation of GLP-1 effect by **9** disappears after washing the cells with assay medium, which demonstrates that the compound does not present a covalent binding.

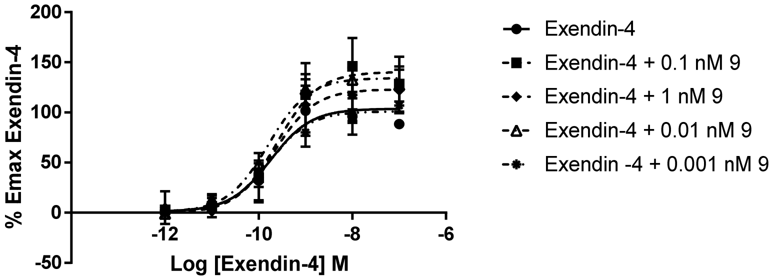

Figure S2. Potentiating effects of **9** on cAMP accumulation on HEK-GLP-1 cells stimulated by increasing doses of GLP-1R agonist exendin-4.

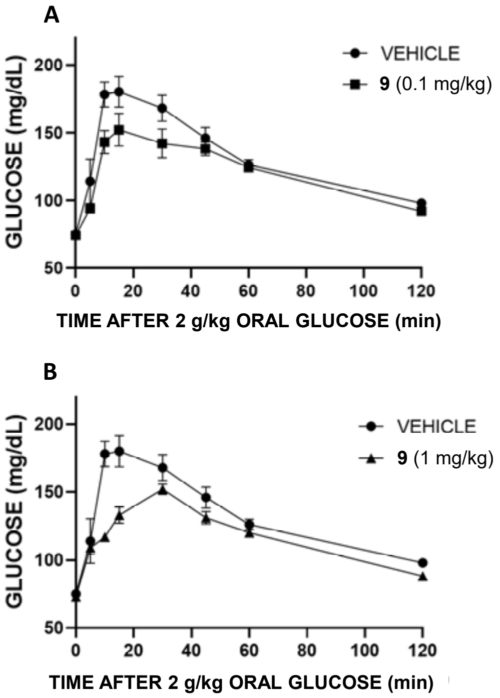

Figure S3. Administration of compound **9** (0.1 (A) or 1 (B) mg/kg, ip) 15 min prior to a glucose (2 g/kg) oral load improved glucose handling in wild-type C57BL/6N male mice. The pharmacological effect was observed basically in the first phase of insulin secretion (0-30 min post-glucose administration), revealing a potentiation of glucose-dependent insulin release. * $P<0.05$ versus vehicle, one ways ANOVA, N=8 animals per group.

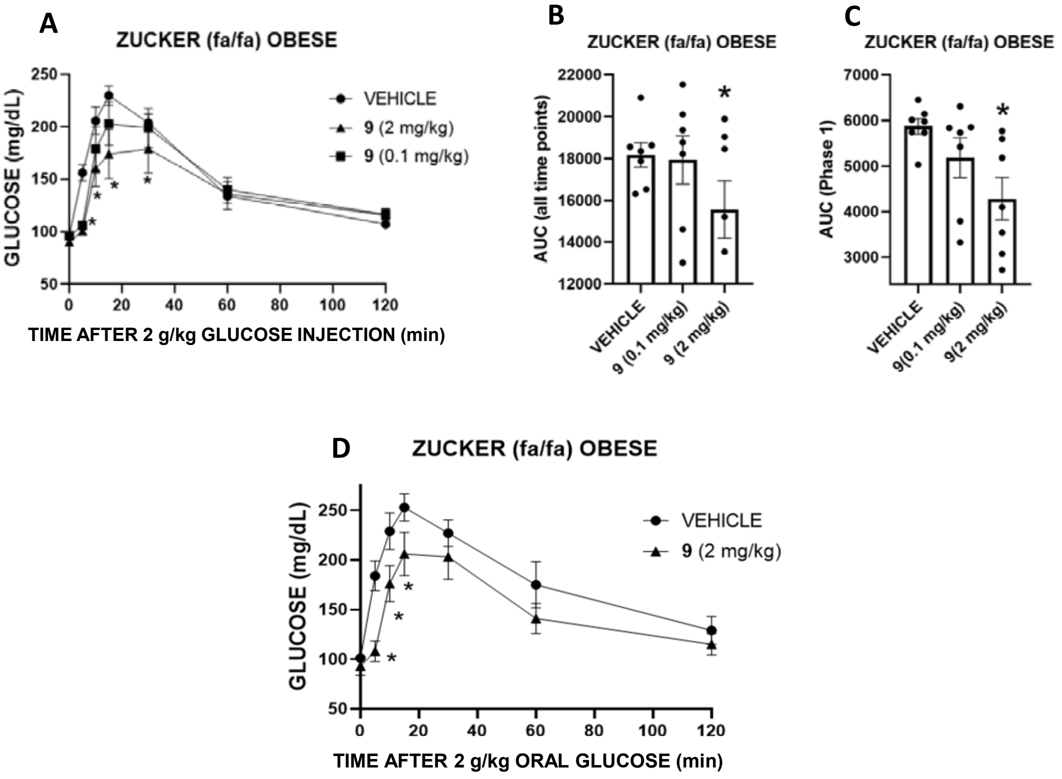

Figure S4. Administration of compound **9** (0.1 or 2 mg/kg, ip) 15 min prior to a glucose (2 g/kg) ip (A) or oral (D) load improved glucose handling in leptin signalling deficient diabetic male Zucker rats. Analysis of the area under curve (AUC, panels B and C) shows how the effect was observed basically in the first phase of insulin secretion (0-30 min post-glucose administration), revealing a potentiation of glucose-dependent insulin release. * $P<0.05$ versus vehicle, one ways ANOVA, N=7-8 animals per group.

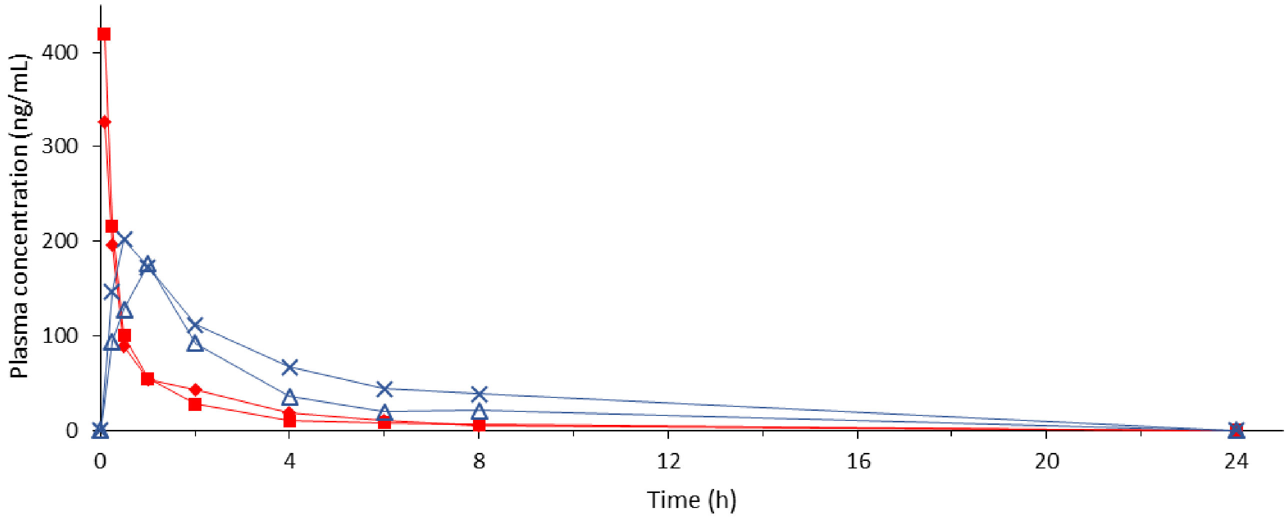

Figure S5. In-vivo pharmacokinetic curves of **9** in rats (two groups of N=4) after oral (blue) and intravenous (red) administration.

A

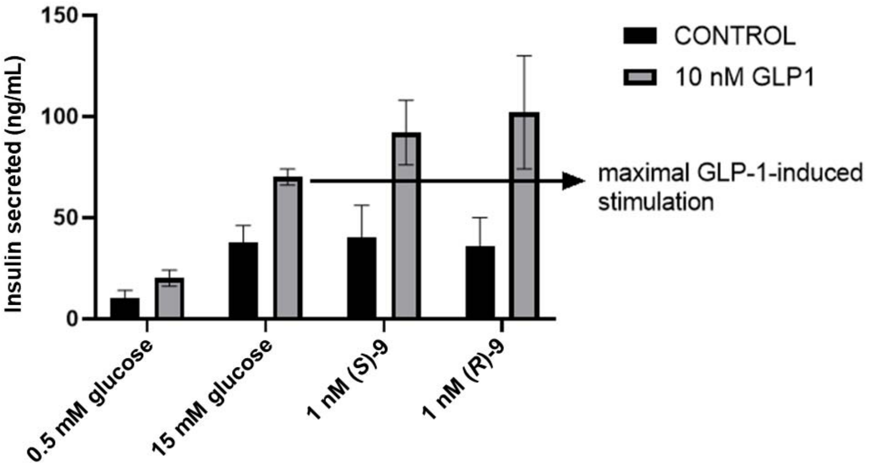

B

Treatment	Insulin secreted (ng/mL)[a]
30 mM KCl	146
0.5 mM Glucose	13
15 mM Glucose	67
0.5 mM Glucose + 0.1 nM GLP-1	25
0.5 mM Glucose + 1 nM GLP-1	28
0.5 mM Glucose + 10 nM GLP-1	34
0.5 mM Glucose + 100 nM GLP-1	32
0.5 mM Glucose + 0.1 nM (*S*)-**9**	29
0.5 mM Glucose + 1 nM (*S*)-**9**	39
0.5 mM Glucose + 10 nM (*S*)-**9**	37
0.5 mM Glucose + 0.1 nM (*R*)-**9**	19
0.5 mM Glucose + 1 nM (*R*)-**9**	31
0.5 mM Glucose + 10 nM (*R*)-**9**	39

[a]Data were obtained from a single representative experiment.

Figure S6. (A) Effects of compounds (*S*)- and (*R*)-**9** on insulin secretion by human pancreatic EndoC-βH1 cells. GLP-1 activated insulin secretion in a glucose-dependent manner ($F(1.16)=17.3$, $p=0.0007$). Both enantiomers display significative activity as potentiators of insulin secretion stimulated by GLP-1 ($F(3.16)=6.9$, $p=0.003$). Data are means ± SEM of three independent measures. (B) Effects of (*S*)- and (*R*)-**9** on insulin secretion by rat INS-1E insulinoma cells. The profile of both enantiomers is similar to that of GLP-1 under low glucose conditions (0.5 mM). Neither GLP-1 nor (*R*)- and (*S*)-**9** reached the insulin secretion produced by high glucose. The

moderate stimulation of insulin secretion observed for (*R*)- and (*S*)-**9** might be attributed to the release of GLP-1 from INS-1E cells.

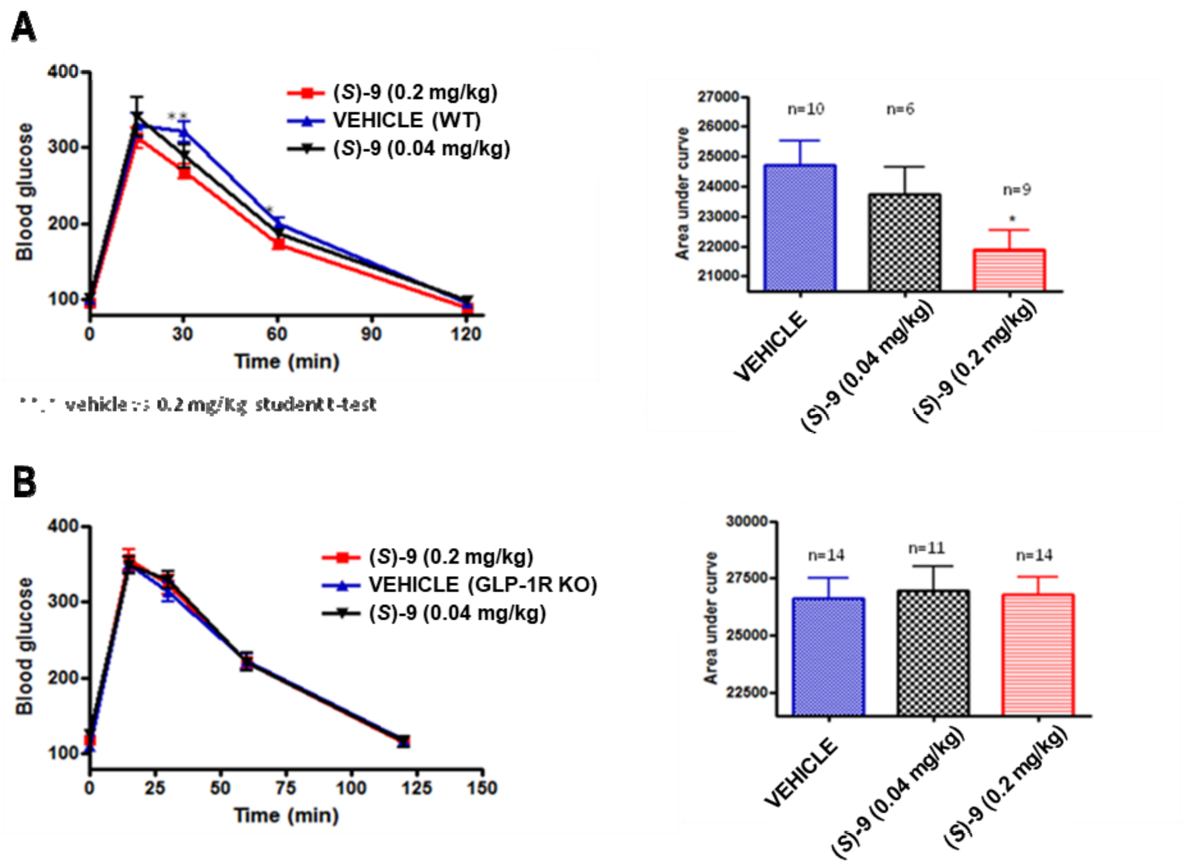

Figure S7. Administration of (*S*)-**9** before a parental load of 2 g/kg glucose induced an improvement of glucose (mg/dL) handling in 12-h fasted wild-type mice (A), when compared to GLP-1R KO mice, where the compound was devoid of effects on insulin handling (B). * *P*<0.05 versus vehicle, one ways ANOVA, N=6-14 animals per group.
